# Rare and Common Genetic Variation Underlying Atrial Fibrillation Risk

**DOI:** 10.1001/jamacardio.2024.1528

**Published:** 2024-06-26

**Authors:** Oliver B. Vad, Laia M. Monfort, Christian Paludan-Müller, Konstantin Kahnert, Søren Z. Diederichsen, Laura Andreasen, Luca A. Lotta, Jonas B. Nielsen, Alicia Lundby, Jesper H. Svendsen, Morten S. Olesen

**Affiliations:** 1 Department of Cardiology, The Heart Centre, Copenhagen University Hospital – Rigshospitalet, Copenhagen, Denmark; 2 Department of Biomedical Sciences, Faculty of Health and Medical Sciences, University of Copenhagen, Copenhagen, Denmark; 3 Department of Clinical Medicine, Faculty of Health and Medical Sciences, University of Copenhagen, Denmark; 4 Regeneron Genetics Center, Tarrytown, New York

## Abstract

**Importance:**

Atrial fibrillation (AF) has a substantial genetic component. The importance of polygenic risk is well established, while the contribution of rare variants to disease risk warrants characterization in large cohorts.

**Objective:**

To identify rare predicted loss-of-function (pLOF) variants associated with AF and elucidate their role in risk of AF, cardiomyopathy (CM), and heart failure (HF) in combination with a polygenic risk score (PRS).

**Design, Setting, and Participants:**

This was a genetic association and nested case-control study. The impact of rare pLOF variants was evaluated on the risk of incident AF. HF and CM were assessed in cause-specific Cox regressions. End of follow-up was July 1, 2022. Data were analyzed from January to October 2023. The UK Biobank enrolled 502 480 individuals aged 40 to 69 years at inclusion in the United Kingdom between March 13, 2006, and October 1, 2010. UK residents of European ancestry were included. Individuals with prior diagnosis of AF were excluded from analyses of incident AF.

**Exposures:**

Rare pLOF variants and an AF PRS.

**Main Outcomes and Measures:**

Risk of AF and incident HF or CM prior to and subsequent to AF diagnosis.

**Results:**

A total of 403 990 individuals (218 489 [54.1%] female) with a median (IQR) age of 58 (51-63) years were included; 24 447 were diagnosed with incident AF over a median (IQR) follow-up period of 13.3 (12.4-14.0) years. Rare pLOF variants in 6 genes (*TTN*, *RPL3L*, *PKP2*, *CTNNA3*, *KDM5B*, and *C10orf71*) were associated with AF. Of these, *TTN*, *RPL3L*, *PKP2*, *CTNNA3*, and *KDM5B* replicated in an external cohort. Combined with high PRS, rare pLOF variants conferred an odds ratio of 7.08 (95% CI, 6.03-8.28) for AF. Carriers with high PRS also had a substantial 10-year risk of AF (16% in female individuals and 24% in male individuals older than 60 years). Rare pLOF variants were associated with increased risk of CM both prior to AF (hazard ratio [HR], 3.13; 95% CI, 2.24-4.36) and subsequent to AF (HR, 2.98; 95% CI, 1.89-4.69).

**Conclusions and Relevance:**

Rare and common genetic variation were associated with an increased risk of AF. The findings provide insights into the genetic underpinnings of AF and may aid in future genetic risk stratification.

## Key Points

**Question:**

What is the combined contribution of rare and common genetic variation to atrial fibrillation (AF) risk?

**Findings:**

In this genetic association study, rare genetic variants, predicted to cause loss of function, in 6 genes were associated with AF. Together, these rare variants and a polygenic risk score for AF were associated with a considerable risk of incident atrial fibrillation; rare variants were also associated with heart failure and cardiomyopathy, and a higher risk of cardiomyopathy following AF diagnosis.

**Meaning:**

The findings suggest that assessing both rare and common genetic variation may aid in atrial fibrillation prevention and risk stratification.

## Teaser

This genetic association study evaluates the association of genetic variants with atrial fibrillation, cardiomyopathy, and heart failure.

## Introduction

Atrial fibrillation (AF) is the most common cardiac arrhythmia, and it is associated with an increased risk of stroke, heart failure (HF), and premature death.^1^ While large genome-wide association studies (GWASs) have uncovered parts of the complex genetic component of AF and identified associations with primarily common genetic variants,^2,3^ it is not always possible to pinpoint a specific causal gene based on the associated locus identified in GWASs.

On the other hand, rare coding variants are in general considered to have large effect sizes on disease risk and prognosis, which might be clinically relevant for the carrier. Several recent studies have suggested genetic testing in some subpopulations of patients with AF (eg, those with early-onset AF).^4,5^ Genetic studies on familial AF have identified associations with several genes, including the ion-channel gene *KCNQ1*^6^ and the sarcomere gene *MYL4*.^7^ However, it is difficult to assess the impact of these variants on AF in the general population, and only a few of these findings have been replicated in large-scale, population-based cohort studies.

By examining whole-exome sequencing data on more than 400 000 individuals, more than 30 000 of whom were diagnosed with AF, we aimed to identify novel gene associations driven by rare variation. Moreover, we aimed to elucidate how such variants influenced risk of incident AF and progression to more severe cardiac disease in combination with polygenic risk. A deeper understanding of genetic causes of AF in the general population may elucidate novel targets for therapeutics and explore the potential of future genetic risk stratification.

## Methods

The study followed the Strengthening the Reporting of Observational Studies in Epidemiology (STROBE) reporting guideline. The study was conducted in the UK Biobank. All participants gave informed written consent. The UK Biobank received ethical approval from North West Multi-Centre Research Ethics Committee. The UK Biobank is a large, population-based cohort study including genetic and clinical information on almost 500 000 individuals representing the general population. The biobank and details on whole-exome sequencing, quality control, and variant annotation have previously been described in detail.^8^ Further filtering, quality control, and phenotype definitions are described in the eAppendix in Supplement 1. A flowchart of participant selection for the study cohort and subsequent analyses is shown in eFigure 1 in Supplement 1.

### Gene-Based Burden Test

Rare coding variants were collapsed into gene-based burden masks. Only variants with minor allele frequencies less than 1% were included in the analyses. Gene masks with a cumulative allele count less than 10 were excluded. In our primary analysis, we focused on rare predicted loss-of-function (pLOF) variants across 17 979 genes. Secondary gene-based tests for association with rare missense variants have been described in the eAppendix in Supplement 1. The burden tests were conducted using the genome-wide regression tests in regenie,^9^ adjusting for age at inclusion, sex, and 10 first principal components. We applied Firth logistic regression when the standard logistic regression *P* was less than .01. We considered associations statistically significant at the *P* < 2.77 × 10^−6^ (corresponding to correction for testing in 18 000 genes). Sensitivity analyses (eAppendix in Supplement 1) included a leave-one-variant-out approach and subgroup analyses without individuals diagnosed with cardiomyopathies at inclusion or during follow-up. We replicated significant associations in an external dataset of 17 910 individuals with AF and 149 348 control individuals (eAppendix in Supplement 1).

For genes in which pLOF variants were significantly associated with AF, we also evaluated the protein and RNA expression. The methodology behind this analysis has been described in detail in the eAppendix in Supplement 1.

### Cardiac *TTN* Isoforms and Exons

The titin protein is expressed in several different isoforms, all encoded by the *TTN* gene.^10^ As a secondary analysis, analyses were conducted, for gene-sets including only the predominant cardiac isoforms of titin (NB2 and NB2A) and constitutively expressed exons (percentage spliced in more than 90% [*TTN*-PSI90]) respectively.

### Aggregate Genetic Risk According to pLOF Variants and Polygenic Risk

To estimate the impact of common genetic variation, we obtained polygenic risk score (PRS) weights from a previously published and validated AF PRS.^11^ The PRS weights were calculated using the PRS–continuous shrinkage (CS)-auto method, and were based on summary statistics from the Atrial Fibrillation Consortium study,^12^ which did not overlap with the UK Biobank. We then calculated PRSs for each individual in the UK Biobank cohort using PLINK^13^ based on the number of risk alleles and the posterior effect size of the variants. The PRS was normalized by scaling to a mean of 0, with an SD equal to 1. Odds ratios (ORs) for AF were calculated per 1-SD increase in PRS using Firth logistic regression models (the logistf packing in R version 1.25.0 [R Foundation]), adjusted for sex, age at inclusion, and principal components 1 to 10. Area under curve (AUC) was estimated using a receiver operator characteristic (ROC) curve.

The PRS was assessed separately in carriers and noncarriers of rare pLOF variants in genes associated with AF (defined as individuals with a pLOF variant in *TTN*, *RPL3L*, *PKP2*, *CTNNA3*, or *KDM5B*). For *TTN*, we only included those with variants in constitutively spliced-in cardiac exons (*TTN*-PSI90). To assess polygenic risk and rare variants in aggregate, the cohort was stratified into groups based on PRS quintile and carrier status for subsequent analyses of OR for AF and absolute risk of AF. To avoid results being driven by relatedness, we conducted a sensitivity analysis on a subset of unrelated individuals (>third degree), described in the eAppendix in Supplement 1.

### Assessment of Risk of Incident AF, Cardiomyopathy, and HF

To assess hazard ratios (HRs) and absolute risk of AF we designed a nested case-control study, with inclusion date as index date and excluding individuals with AF, HF, or cardiomyopathy diagnosed prior to inclusion. The cohort was stratified into 10 groups based on PRS and carrier status of pLOF variants, as described above. HRs were calculated using a Cox regression model and adjusted for sex, age at inclusion, and clinical risk factors at baseline (obesity, hypertension, ischemic heart disease [IHD], and HF). Individuals were followed up with until the date of AF diagnosis and censored at death or end of follow-up (July 1, 2022), whichever came first. Obesity was defined as body mass index (BMI) of 30 or greater (calculated as weight in kilograms divided by height in meters squared) at inclusion. Other phenotype definitions are described in the eAppendix in Supplement 1.

Crude cumulative incidences were estimated using the Aalen-Johansen estimator (prodlim package in R version 2019.11.13), accounting for all-cause mortality as a competing risk. Models were constructed as time-to-event analyses, with date of inclusion as index date and age in years as the time scale. We then further stratified by age groups (above or below 60 years at inclusion) and estimated 10-year risk of AF, using time since inclusion as the time scale. To assess the interplay between polygenic risk and clinical risk factors, another model was constructed by stratifying the cohort based on these age groups and 2 common and modifiable risk factors for AF: obesity and hypertension.^14^ In sensitivity analyses, we examined a subset of unrelated individuals and investigated models without *TTN* variants among the pLOF variants.

Using cause-specific Cox regressions, we evaluated how genetic predisposition to AF influenced the risk of HF and cardiomyopathy. Genetic predisposition was represented by all pLOF variants in the 5 genes described above; all pLOF variants, excluding those in *TTN*; and the PRS for AF (per SD increase). The methodology behind these analyses is described in detail in the eAppendix in Supplement 1.

## Results

We conducted gene-based association tests across the exome of 403 990 individuals. Baseline characteristics of the cohort are summarized in Table 1. The median (IQR) age at inclusion was 58 (51-63) years, and 218 489 participants (54.1%) were female. A total of 6677 individuals had an AF diagnosis at inclusion in the biobank, and 24 447 individuals were diagnosed with AF by the end of follow-up. The 31 124 individuals with a diagnosis of AF were defined as cases in the gene-based burden tests, while the remaining 372 871 individuals were considered controls.

**Table 1.** Baseline Characteristics at Inclusion

UK Biobank cohort (N = 403 990)	No. (%)
Age, median (IQR), y	58 (51-63)
Sex	218 489 (54.1)
Female	218 489 (54.1)
Male	218 489 (54.1)
BMI, mean (SD)	27.4 (4.8)
Atrial fibrillation	6677 (1.7)
Hypertension	107 905 (26.7)
Heart failure	2252 (0.6)
Ischemic heart disease	21 957 (5.4)
Stroke	6626 (1.6)
Diabetes	18 473 (4.6)

Abbreviation: BMI, body mass index (calculated as weight in kilograms divided by height in meters squared).

### Genetic Association With pLOF Variants in 6 Genes

We identified significant associations between AF and pLOF variants in the genes *PKP2* (OR, 2.12; 95% CI, 1.60-2.82; *P* = 2.21 × 10^−07^), *CTNNA3* (OR, 2.79; 95% CI, 1.88-4.14; *P* = 3.74 × 10^−07^), *C10orf71* (OR, 2.33; 95% CI, 1.69-3.39; *P* = 7.83 × 10^−07^), and *KDM5B* (OR, 2.70; 95% CI, 1.80-4.06; *P* = 1.76 × 10^−06^) and replicated the previously reported association between AF and pLOF variants in the genes *TTN* (OR, 1.77; 95% CI, 1.60-1.95; *P* = 3.38 × 10^−30^)^15^ and *RPL3L* (OR, 1.56; 95% CI, 1.38-1.77; *P* = 1.69 × 10^−12^).^16^ A Manhattan plot and quantile-quantile plot of associations are provided in eFigures 2 and 3 in Supplement 1. Significant genetic associations are summarized in Table 2.

**Table 2.** Associations Between Atrial Fibrillation (AF) and Rare Predicted Loss-of-Function (pLOF) Variants in Gene-Based Burden Tests^a^

Gene	Protein	OR (95% CI)	*P* value
*TTN* (PSI90)	Titin (constitutively expressed cardiac exons [spliced in >90%])	3.85 (3.25 to 4.55)	1.09 × 10^−55^
*TTN* (N2BA-N2B)	Titin (cardiac isoforms)	2.17 (1.93 to 2.43)	2.07 × 10^−40^
*TTN*	Titin	1.77 (1.60 to 1.95)	3.38 × 10^−30^
*RPL3L*	Ribosomal protein L3-like	1.56 (1.38 to 1.77)	1.69 × 10^−12^
*PKP2*	Plakophilin 2	2.12 (1.60 to 2.82)	2.21 × 10^−07^
*CTNNA3*	Catenin α3	2.79 (1.88 to 4.14)	3.74 × 10^−07^
*C10orf71*	Cardiac-enriched *FHL2*-interacting protein	2.40 (1.69 to 3.39)	7.83 × 10^−07^
*KDM5B*	Lysine demethylase 5B	2.70 (1.80 to 4.06)	1.76 × 10^−06^

Abbreviation: OR, odds ratio.

^a^ *P* values <2.77 × 10^−6^ were considered exomewide significant. *TTN* (N2BA-N2B), test restricted to cardiac isoforms of *TTN* gene; *TTN* (PSI90), test restricted to constitutively expressed cardiac exons in *TTN* gene (percent spliced in >90%).

Full results from the gene-based burden test of pLOF variants have been summarized in eTable 10 in Supplement 2. Protein and RNA expression analyses revealed that the associated genes were predominantly expressed in cardiomyocytes, except *KDM5B*, which was expressed across all cell types (eAppendix and eFigure 4 in Supplement 1). As the *C10orf71* gene had not previously been associated with cardiovascular disease, we examined tissue-specific expression of the gene and found that it was almost exclusively expressed in muscle tissue (eFigure 5 in Supplement 1). In an external replication cohort of 17 910 individuals with AF and 149 348 control individuals, we found replicated associations for *TTN*, *PKP2*, *CTNNA3, RPL3L*, and* KDM5B* (eAppendix and eFigure 6 in Supplement 1). The variants contributing to each gene mask are summarized in eTable 11 in Supplement 2.

Secondary analyses on pLOF variants in *TTN* cardiac isoforms (N2BA and N2B) and constitutively expressed cardiac exons found an even greater OR for AF (OR, 2.17; 95% CI, 1.93-2.43; *P* = 2.07 × 10^−40^ and OR, 3.85; 95% CI, 3.25-4.55; *P* = 1.09 × 10^−55^). Secondary gene-based tests for rare missense variants replicated one previously known association with the gene *UBE4B* (OR, 1.22; 95% CI, 1.12-1.31; *P* = 5.90 × 10^−7^) (eTables 11 and 12 in Supplement 2). Sensitivity analyses did not substantially alter the results, except for pLOF variants in *RPL3L* and missense variants in *UBE4B* (eAppendix and eTables 4-7 in Supplement 1). We note that for *RPL3L *the association was predominantly driven by a pLOF variant, while for *UBE4B* it was driven by a missense variant.

### Aggregate Genetic Risk According to pLOF Variants and Polygenic Risk

We evaluated polygenic risk using an externally derived PRS. A 1-SD increase in this PRS was associated with an OR of 1.53 (95% CI, 1.51-1.55; *P* < .001) for AF. The addition of the PRS yielded an AUC of 0.76 (95% CI, 0.75-0.76) compared to an AUC of 0.74 (95% CI, 0.74-0.74) in a reference model adjusted for sex, age at inclusion, and principal components 1 to 10.

**Figure 1.**
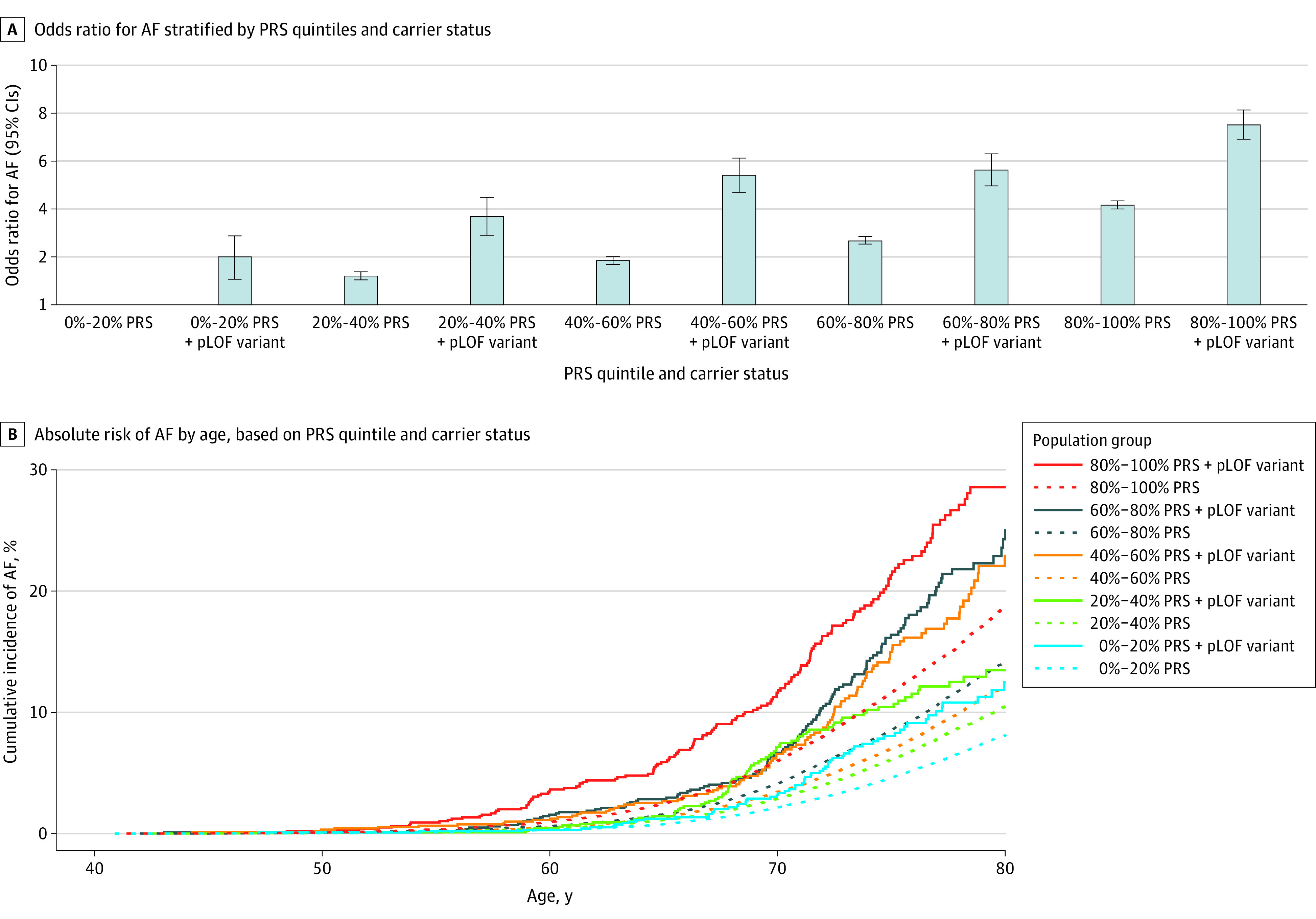
Risk of Atrial Fibrillation (AF) According to Polygenetic Risk Score (PRS) and Rare Variants A, Odds ratios for AF in UK Biobank cohort are stratified by PRS quintile and carrier status of rare predicted loss-of-function (pLOF) variants in AF-associated genes. The group with a low PRS (0%-20%) and no pLOF variant is considered as the reference, with the base line representing the reference odds ratio of 1.0. B, Absolute risk of AF in shown by age, based on PRS quintile and carrier-status of rare pLOF variants in AF-associated genes. Date of inclusion was used as the index date.

A 1-SD increase in PRS was associated with elevated OR estimates for AF in carriers of rare pLOF variants (OR, 1.69; 95% CI, 1.54-1.85; *P* < .001) compared with noncarriers (OR, 1.53; 95% CI, 1.51-1.55; *P* < .001). When assessing PRSs and rare pLOF variants in aggregate, we observed a dose-response–like increase in OR from the group with lowest genetic risk to the group with the highest risk. The group with a PRS in the top quintile and a rare pLOF variant had a markedly increased OR for AF of 7.08 (95% CI, 6.03-8.28) compared with noncarriers with low PRSs. Results have been illustrated in Figure 1A and summarized in eTable 1 in Supplement 1. Similar estimates were observed in a subset of unrelated individuals (eTable 2 in Supplement 1).

### Incident AF According to Combined Genetic Risk

After excluding individuals with prevalent AF, HF, or cardiomyopathy at baseline, 5032 individuals (1.27%) were carriers of a rare pLOF variant (eTable 3 in Supplement 1). During a median (IQR) follow-up period of 13.3 (12.4-14.0) years, 24 061 individuals were diagnosed with incident AF, while 23 907 died before AF diagnosis or end of follow-up. The group with both a PRS in the top quintile and a rare pLOF variant had an HR of 4.78 for incident AF (95% CI, 4.06-5.63; *P* < .001) compared with noncarriers with low PRSs. Estimates for all model covariates are provided in eTable 4 in Supplement 1.

This trend was also observed for an absolute risk of AF, where pLOF variant carriers with a high PRS had a cumulative AF incidence of 28.55% (95% CI, 24.5-33.2) by age 80 years (Figure 1B). Comparatively, noncarriers with middle (40%-60%) and low (0%-20%) PRSs, had absolute risks of 12.1% (95% CI, 11.7-12.5%) and 8.1% (95% CI, 7.8-8.4%), respectively (eTables 5-7 in Supplement 1). Sensitivity analyses in unrelated individuals and models excluding pLOF variants in *TTN* did not substantially alter the results (eTables 8 and 9 in Supplement 1).

**Figure 2.**
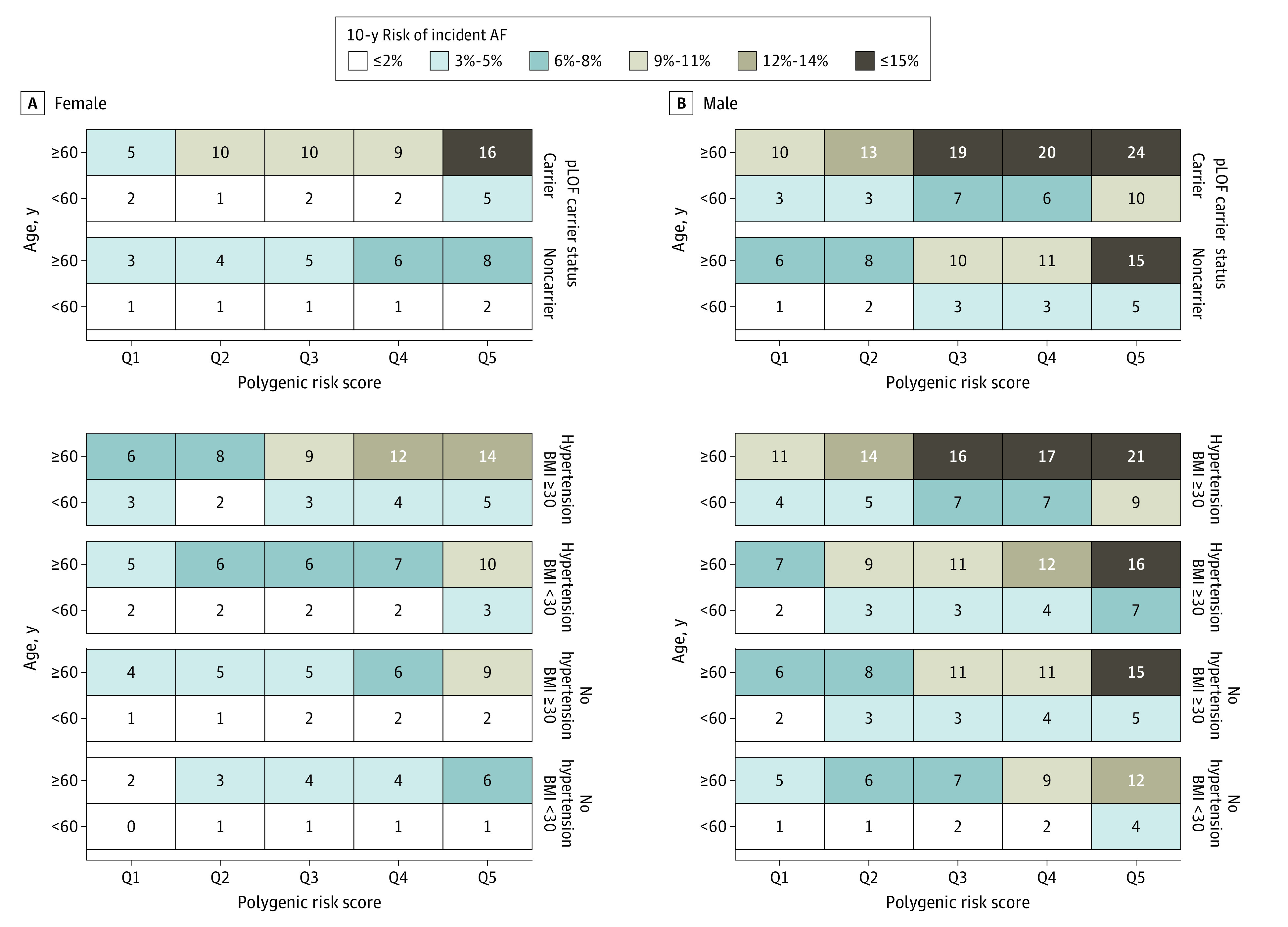
Risk of Atrial Fibrillation (AF) over 10 Years Absolute 10-year risk of AF by sex. The top 2 subpanels show the 10-year risk of AF for individuals with and without a predicted loss-of-function (pLOF) variant in AF-associated genes. The bottom 4 subpanels show the 10-year risk of AF in individuals according to 2 common risk factors: obesity and hypertension. The individual tiles denote absolute risk of AF within 10 years. Q indicates quintile.

Concordant results were observed in analyses on 10-year absolute risk of AF (Figure 2). Individuals with a high PRS for AF consistently had a higher 10-year risk of AF across age groups and sex. The risk of AF was more pronounced in individuals with BMI of 30 or greater and hypertension. The highest risk was found in individuals older than 60 years at inclusion with high PRSs who also carried a rare pLOF variant (15% and 25% in female individuals and male individuals, respectively). Sensitivity analyses in a subset of unrelated individuals (>third degree) and models not considering *TTN* pLOF variants did not substantially alter these estimates (eFigures 7 and 8 in Supplement 1).

### Genetic Predisposition for AF and Risk of HF and Cardiomyopathy

When regarding AF, cardiomyopathy, and HF as competing events, rare pLOF variants in AF-associated genes conferred an increased HR for incident AF (HR, 1.85; 95% CI, 1.69-2.02; *P* < .001), incident cardiomyopathy (HR, 3.13; 95% CI, 2.24-4.36; *P* < .001), and incident HF (HR, 1.51; 95% CI, 1.26-1.82; *P* < .001). When not considering *TTN* variants, the effect estimates for rare pLOF variants were attenuated for AF (HR, 1.60, 95% CI, 1.44-1.78; *P* < .001) and no longer statistically significant for cardiomyopathy (HR, 1.39; 95% CI, 0.80-2.40; *P* = .24) and HF (HR, 1.01; 95% CI, 0.79-1.29, *P* = .95). The AF PRS was associated with AF (HR per SD, 1.45; 95% CI, 1.43-1.47; *P* < .001) but not with cardiomyopathy (HR per SD, 0.99; 95% CI, 0.92-1.06; *P* = .73) or HF (HR per SD, 1.02; 95% CI, 0.99-1.05; *P* = .12). Sensitivity analyses in a subset of unrelated individuals showed similar results (eFigure 9 in Supplement 1).

**Figure 3.**
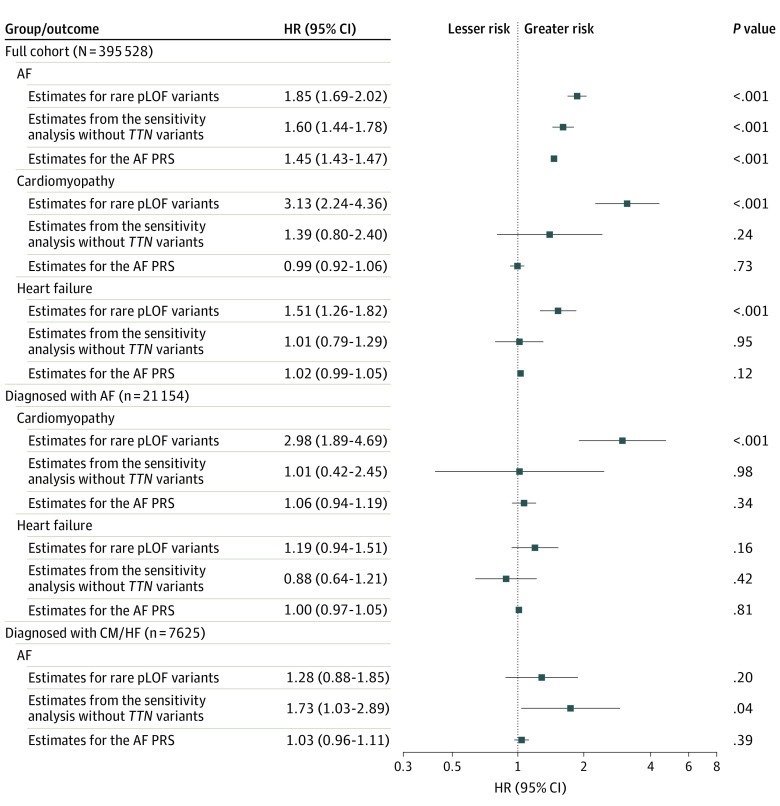
Incident Atrial Fibrillation (AF), Cardiomyopathy, and Heart Failure According to Rare and Common Genetic Variation Estimates from cause-specific Cox regressions according to predisposition for AF. First-incident AF, cardiomyopathy, heart failure, and all-cause mortality were considered competing events. The figure shows results from the full cohort and subgroup analyses on individuals diagnosed with AF, or cardiomyopathy or heart failure during follow-up.

We identified 21 154 individuals diagnosed with AF after inclusion into UK Biobank, and no prior diagnosis of HF or cardiomyopathy. Regarding cardiomyopathy and HF as competing events, we observed an increased HR for cardiomyopathy in carriers of rare pLOF variants in AF-associated genes (HR, 2.98; 95% CI, 1.89-4.69; *P* < .001). We did not observe significant effects of pLOF variants when excluding *TTN* variants (HR, 1.01; 95% CI, 0.42-2.45; *P* = .98) or for the AF PRS (HR per SD, 1.06; 95% CI, 0.94-1.19; *P* = .34). We examined the risk of incident AF in another subgroup of 7625 individuals with incident cardiomyopathy or HF during follow-up, without a prior diagnosis of AF. Here, we found no significant associations between pLOF variants or AF PRS and incident AF, although we noted increased estimates in carriers of pLOF variants in AF-associated genes excluding *TTN* (HR, 1.73; 95% CI, 1.03-2.89; *P* = .04). Sensitivity analyses in unrelated individuals and the application of a 30-day grace period did not substantially alter the results (eFigure 10 in Supplement 1). All estimates are shown in Figure 3.

## Discussion

In this genetic association study, we examined large-scale, whole-exome sequencing data on more than 400 000 individuals in the UK Biobank, including approximately 31 000 individuals with AF. Whole-exome sequencing of large population-based cohorts offers several advantages compared to former genetic approaches. The method enables identification of rare coding variants with large effect sizes that are often not picked up with GWAS genotyping. Using a gene-based burden test, we identified several genes in which rare pLOF variants conveyed a considerably increased OR for AF. We demonstrated that polygenetic risk of AF and rare pLOF variants were associated with an increased absolute risk of incident AF and investigated the combined effect of an AF PRS and clinical risk factors (obesity and hypertension). Individuals with a pLOF variant had a substantial absolute risk of AF comparable to those with both BMI of 30 or greater and hypertension. Consequently, our results indicate a benefit in including both common and rare genetic variation in AF risk stratification.

Our study identified several novel associations between pLOF variants and with AF in key genes in ventricular cardiomyopathies, as well as genes not previously linked with heart disease. Among these were novel associations with the genes *CTNNA3* and *KDM5B*. The *CTNNA3* gene encodes the cytoskeletal protein catenin α3, which interacts with cardiomyocyte desmosomes and plays a role in cell adhesion.^17^ Variants in *CTNNA3* have been putatively associated with arrhythmogenic right ventricle cardiomyopathy^18^ and rare variants in *CTNNA3* have also been associated with familial AF.^19^ The *KDM5B* gene has not previously been associated with arrhythmias. However, the gene encodes lysine-specific demethylase 5B, a protein involved in histone methylation. This protein is thought to play a role in cardiac fibrosis,^20^ a common substrate in reentry arrhythmia mechanisms. We also replicated an association with pLOF variants in the *RPL3L* gene, which is involved in ribosomal function and muscle growth.^21^ Sensitivity analyses revealed that the association was primarily driven by a splice-donor variant, which has previously been reported in an AF GWAS.^16^

Results also showed an association with the *C10orf71* gene that did not replicate in the external dataset. While a locus near the *C10orf71* gene was recently associated with AF,^22^ knowledge of the *C10orf71* gene and the functions of the encoded protein cardiac-enriched FHL2-interacting protein (CEFIP) is relatively sparse. One study^23^ has suggested that the protein may locate to sarcomere Z-discs and contribute to the regulation of cardiomyocyte hypertrophy, which is consistent with proteomics and single-cell sequencing data showing expression in both atria, with a predominant expression in cardiomyocytes. As the genetic association did not externally replicate, its potential role in AF remains uncertain until validated in other studies.

*TTN* is a well-established dilated cardiomyopathy gene,^24^ and its association with AF has been hypothesized to be partly driven by an atrial cardiomyopathy.^15^ Interestingly, we also identified an AF association with pLOF variants in the *PKP2* gene, which plays a major role in arrhythmogenic right ventricle cardiomyopathy.^25^ Common variants in a locus near *PKP2* have previously been associated with AF in GWASs, and our study corroborates these findings with evidence of a role of pLOF variants in *PKP2* in AF.

Given the discovered associations with several cardiomyopathy genes and previous reports of increased mortality in rare variant carriers,^5^ we examined whether carriers of rare pLOF variants in AF-associated genes were at a greater risk of cardiomyopathy or HF. AF and HF often coexist, and their temporal relationship can be complex and challenging to disentangle.^26^ In an effort to elucidate the role of genetic variation in this context, we conducted cause-specific Cox regressions with AF, HF, and cardiomyopathy as competing events. We found that both rare pLOF variants and the AF PRS associated with incident AF prior to a potential diagnosis of HF or cardiomyopathy. Genetic predisposition for AF was also associated with incident cardiomyopathy or HF prior to AF, where we noted high HRs for cardiomyopathy in pLOF variant carriers. Moreover, rare pLOF variants were associated with an increased risk of cardiomyopathy in individuals with AF but not vice versa. These findings indicate that for some variant carriers, AF may be the first disease manifestation preceding more severe cardiac disease.

While the prevalence of rare pLOF variants is relatively low in the general population, their contribution to disease risk and their associations with more severe cardiac disease may justify genetic testing in specific patient groups.^27^ Previous research has shown a higher proportion of rare variants in younger patients with AF^4^ and found an increased mortality in variant carriers.^5^ Based in part on these findings, recent guidelines on AF from the American College of Cardiology and American Heart Association^28^ suggest that genetic testing or surveillance for cardiomyopathy may be reasonable in patients with AF with onset before 45 years of age. Our findings align with these recommendations and may contribute to a better understanding of specific genes in which rare pLoF variants convey a large risk of AF. Although population-wide genetic screening for rare variants is unlikely in the near future, integrating genetic perspectives may aid in AF risk stratification as sequencing becomes cheaper and more readily available. Future studies focused on identifying patient or population groups that may benefit from genetic testing are warranted.

### Limitations

The reported results should be interpreted with respect to the study limitations. First, in order to avoid bias from population stratification, we only included individuals of European ancestry and our results may therefore not be generalizable to other populations. Second, correcting for thousands of independent tests led us to apply a strict significance threshold, which may have limited our power to detect potential genes associated with AF. Third, the gene-based association tests and analyses on absolute risk of AF were observational findings. Hence, they may be biased by residual confounding and confounding by indication and cannot be assumed to represent causal relationships. Although 5 of the 6 associated genes were replicated in an external cohort, they should also be validated in functional studies and in other cohorts and population groups to better understand the pathophysiological mechanisms leading to arrhythmia. Additionally, it should be noted that the study design of the UK Biobank may also introduce healthy volunteer bias.

## Conclusions

In summary, our study identified novel genetic associations with AF, including several hallmark genes of major cardiomyopathy subtypes. The genes identified were involved in diverse cellular processes, including sarcomere and desmosome structure, while the associations with genes involved in ribosomal function, histone methylation and ubiquitination hint at novel arrhythmia mechanisms. We showed an interplay between rare and common genetic variation and demonstrated a substantial absolute risk of AF in individuals with a high PRS carrying a rare pLOF variant. These findings may contribute to possible future genetic risk stratification and improved clinical practice.

## Notes

Supplement 1. eAppendix eTable 1. Odds ratio for AF according to PRS and pLOF variants eTable 2. Odds ratio for AF according to PRS and pLOF variants (unrelated individuals) eTable 3. Variant carriers in study cohort for incident AF, HF and cardiomyopathy eTable 4. Hazard ratios ratio for incident AF according to genetic risk and clinical risk factors eTable 5. Cumulative incidence of AF by age 80 eTable 6. Cumulative incidence of AF by age 70 eTable 7. Cumulative incidence of AF by age 60 eTable 8. Cumulative incidence of AF by age 80 (unrelated individuals) eTable 9. Cumulative incidence of AF by age 80 (excluding TTN pLOF variants) eFigure 1. Flowchart of study design eFigure 2. Manhattan plot of gene-based test for rare pLOF variants eFigure 3. Quantile-Quantile plot of gene-based test for rare pLOF variants eFigure 4. Cardiac expression of AF associated genes eFigure 5. Tissue-specific RNA expression of C10orf71 eFigure 6. Results from gene-based association test with AF in independent replication cohort eFigure 7. Ten-year risk of AF (unrelated individuals) eFigure 8. Ten-year risk of AF (excluding TTN pLOF variants) eFigure 9. Forest plot of hazard ratios for AF, cardiomyopathy, and HF (unrelaed individuals) eFigure 10. Forest plot of hazard ratios for AF, cardiomyopathy, and HF (30-day grace period) eReferences

Supplement 2. eTable 10. Gene-based burden test (pLOF variants) eTable 11. Variants in gene masks (pLOF variants) eTable 12. Gene-based burden test (missense variants, additive model) eTable 13. Gene-based burden test (missense variants, ACATO model) eTable 14. LOVO analysis (pLOF variants) eTable 15. LOVO analysis (missense variants) eTable 16. Gene-based burden test, excluding cardiomyoopathy (pLOF variants) eTable 17. Gene-based burden test, excluding cardiomyopathy (missense variants)

Supplement 3. Geisinger MyCode Community Health Initiative and Regeneron Genetics Center (RGC) Research Team members

Supplement 4. Data sharing statement
