# Supplementary material for: Rare and Common Genetic Variation Underlying Atrial Fibrillation Risk
Source: JAMA Cardiol. 2024 Jun 26;9(8):732–40. doi: 10.1001/jamacardio.2024.1528 (PMC11209175; doi:10.1001/jamacardio.2024.1528)
Supplement: Supplement 1. — eAppendix eTable 1. Odds ratio for AF according to PRS and pLOF variants eTable 2. Odds ratio for AF according to PRS and pLOF variants (unrelated individuals) eTable 3. Variant carriers in study cohort for incident AF, HF and cardiomyopathy eTable 4. Hazard ratios ratio for incident AF according to genetic risk and clinical risk factors eTable 5. Cumulative incidence of AF by age 80 eTable 6. Cumulative incidence of AF by age 70 eTable 7. Cumulative incidence of AF by age 60 eTable 8. Cumulative incidence of AF by age 80 (unrelated individuals) eTable 9. Cumulative incidence of AF by age 80 (excluding TTN pLOF variants) eFigure 1. Flowchart of study design eFigure 2. Manhattan plot of gene-based test for rare pLOF variants eFigure 3. Quantile-Quantile plot of gene-based test for rare pLOF variants eFigure 4. Cardiac expression of AF associated genes eFigure 5. Tissue-specific RNA expression of C10orf71 eFigure 6. Results from gene-based association test with AF in independent replication cohort eFigure 7. Ten-year risk of AF (unrelated individuals) eFigure 8. Ten-year risk of AF (excluding TTN pLOF variants) eFigure 9. Forest plot of hazard ratios for AF, cardiomyopathy, and HF (unrelaed individuals) eFigure 10. Forest plot of hazard ratios for AF, cardiomyopathy, and HF (30-day grace period) eReferences [file jamacardiol-e241528-s001.pdf]

Supplemental Online Content

Vad OB, Monfort LM, Paludan-Müller C, et al; the Geisinger MyCode Community Health Initiative, Regeneron Genetics Center. Rare and common genetic variation underlying atrial fibrillation risk. *JAMA Cardiol*. Published online June 26, 2024. doi:10.1001/jamacardio.2024.1528

eAppendix

eTable 1. Odds ratio for AF according to PRS and pLOF variants

eTable 2. Odds ratio for AF according to PRS and pLOF variants (unrelated individuals)

eTable 3. Variant carriers in study cohort for incident AF, HF and cardiomyopathy

eTable 4. Hazard ratios ratio for incident AF according to genetic risk and clinical risk factors

eTable 5. Cumulative incidence of AF by age 80

eTable 6. Cumulative incidence of AF by age 70

eTable 7. Cumulative incidence of AF by age 60

eTable 8. Cumulative incidence of AF by age 80 (unrelated individuals)

eTable 9. Cumulative incidence of AF by age 80 (excluding TTN pLOF variants)

eFigure 1. Flowchart of study design

eFigure 2. Manhattan plot of gene-based test for rare pLOF variants

eFigure 3. Quantile-Quantile plot of gene-based test for rare pLOF variants

eFigure 4. Cardiac expression of AF associated genes

eFigure 5. Tissue-specific RNA expression of C10orf71

eFigure 6. Results from gene-based association test with AF in independent replication cohort

eFigure 7. Ten-year risk of AF (unrelated individuals)

eFigure 8. Ten-year risk of AF (excluding TTN pLOF variants)

© 2024 Vad OB et al. *JAMA Cardiology.*

eFigure 9. Forest plot of hazard ratios for AF, cardiomyopathy, and HF (unrelaed individuals)

eFigure 10. Forest plot of hazard ratios for AF, cardiomyopathy, and HF (30-day grace period)

eReferences

This supplemental material has been provided by the authors to give readers additional information about their work.

© 2024 Vad OB et al. *JAMA Cardiology.*

Quality control, variant annotation, and phenotype definitions

We conducted further filtering of samples based on QC criteria listed in UK Biobank resource 531 (heterozygosity, missing rates, excess relatedness, and missing kinship inference). We excluded samples with disagreements between reported sex and genetically determined sex, and filtered for European ancestry based on the first six principal components of individuals self-reporting as "White", "Irish", or "Any other white background" (UK Biobank data field 21000, coding 1001, 1002, and 1003). We filtered variants by missingness (>10%) and Hardy-Weinberg equilibrium test ($P<1x10^{-15}$), and retained calls with a genotype quality >20, read depth >10 and call rate >90%.

Variant annotation was performed using dbSNP (version 4.1a)[1] and SnpEff (version 5.0)[2]. pLoF variants were defined as variants leading to a premature stop codon or to the loss of a start or stop codon, frameshift variants leading to a premature stop codon, and variants disrupting canonical splice acceptor or donor sites. Only pLoF variants annotated as "high" impact were included as pLoF variants. We assesed splice-sit variants using SpliceAI[3], and classified splice-site variants with SpliceAI score >0.8 as pLoF.

AF was defined by the *International Classification of Diseases, 10th revision* (ICD-10) code I48, corresponding to UK Biobank data field 131351. The AF diagnosis in the UK Biobank was based on hospital records, death records and primary care records. Individuals without an AF diagnosis were used as controls. Individuals with uncertain AF diagnosis (i.e. individuals with AF diagnosis based only on self-reports or individuals diagnosed with atrial flutter [ICD-10 code I48.3 and I48.4]) were assigned to the control group.

Heart failure was defined by ICD-10 code I50 (UK Biobank data field 131354) and cardiomyopathy by ICD10-code I42 (UK Biobank data field 131338).

Ischemic heart disease was defined by ICD-10 codes I20, I21, I22, I24, and I25 (UK Biobank data fields 131296, 131298, 131300, 131304, and 131306).

Hypertension was defined by ICD-10 code I10 (UK Biobank data field 131286) diagnosed at time of inclusion.

© 2024 Vad OB et al. *JAMA Cardiology.*

Diabetes was defined by ICD-10 codes E10, E11 and E14 (UK Biobank data fields f.130706, f.130708, and f.130714)

Gene-based tests for rare missense variants

Unlike pLOF variants, the effects of missense variants on disease risk are more difficult to predict. Traditional burden tests lose power when the effects of variants are bidirectional. Alternative methods that account for bi-directionality, like the Sequence Kernel Association Test (SKAT)[4], may lose power when only a small proportion of variants in a gene are associated with the investigated outcome (sparsity of causal variants).

We, therefore, performed both a traditional burden test and followed up with the Omnibus Aggregated Cauchy Association Test (ACAT-O)[5] as a sensitivity analysis. The ACAT-O test is robust to both bi-directional variant effects and sparsity of causal variants and is therefore well-suited to examine missense variants.

We identified one exome-wide significant association with rare missense variants in the gene *UBE4B* (OR: 1.22; P=5.90x10^{-7}). Using the ACAT-O approach which is more robust to bidirectional variant effects, we again found an association with the *UBE4B* gene (P=4.85x10^{-11}), as well as the *FAM189A2* gene (P=5.78x10^{-7}). Results from the missense variant analyses are summarized in **Supplementary Data 2-3**.

Sensitivity analyses for gene-based tests

As a sensitivity analysis, we conducted a leave-one-variant-out (LOVO) analysis using the function integrated in REGENIE, for all significant and suggestive associations. This approach constructs a series of masks for each gene, leaving one variant out per mask. A subsequent gene-based burden test was performed for each mask to detect if any individual variants were the sole drivers of the association (P>0.05 for mask without individual variant).

To assess whether any associations were primarily driven by ventricular cardiomyopathies, we also conducted another gene-based burden test, excluding all individuals with diagnosed

© 2024 Vad OB et al. *JAMA Cardiology.*

cardiomyopathies before inclusion or during follow-up. Cardiomyopathies were defined by ICD10 code I42 (UK Biobank data field 131338).

The association between pLOF variants in *RPL3L* and AF was primarily driven by a variant in position chr16:1945498:C>T (P=0.050 for mask without variant) and the association between missense variants in the *UBE4B* gene and AF was primarily driven by a single missense variant in position chr1:10107367:G>A (P=0.74 for mask without variant). Results of the LOVO analysis are summarized in **Supplementary Data 4-5**. Excluding individuals with cardiomyopathies did not substantially alter the results (**Supplementary Data 6-7**).

Replication of genetic findings

We included 138,131 participants in Geisinger Health System MyCode cohort and 29,127 participants in the Mount Sinai BioMe Biobank. Atrial fibrillation cases were defined based on International Classification of Diseases version 10 (ICD-10) I48 obtained from electronic health records. Participants without any records of cardiac arrythmia were used as controls.

DNA sequencing and genotyping data

The Regeneron Genetics Center performed high coverage whole-exome sequencing using NimbleGen VCRome probes (Roche CA, USA) or a modified version of the xGen design from Integrated DNA Technologies (IDT). Sequencing was done using Illumina v4 HiSeq 2500 or NovaSeq instruments, achieving over 20x coverage for 96% of VCRome samples and 99% of IDT samples. Variants were annotated using snpEff and Ensembl v85 gene definitions, prioritizing protein-coding transcripts based on functional impact. The following variants were defined as protein truncating: insertions or deletions resulting in frameshift, any variant causing a stop gained, start lost or stop lost and any variants affecting a splice acceptor or splice donor site. Common variant genotyping was performed on single nucleotide polymorphism (SNP) arrays as previously described[6]. We retained genotyped variants with a minor allele frequency >1%, <10% missingness, Hardy-Weinberg equilibrium test P-value >10-15. We imputed the genotyped variants based on the TOPMed reference panel[7], using the TOPMed imputation server[8,9]. Further details are provided elsewhere[6,10,11].

© 2024 Vad OB et al. *JAMA Cardiology.*

Association analyses

We estimated associations between the burden of predicted loss-of-function variants in TTN, RPL3L, PKP2, CTNNA3, C10orf71, and KDM5B with atrial fibrillation by fitting additive genetic Firth bias-corrected logistic regression models using the software REGENIE, version 2+[12]. Analyses were adjusted for age, age squared, sex, age-by-sex, and age squared–by–sex interaction terms; experimental batch-related covariates; the first 10 common variant–derived genetic principal components; the first 20 rare variant–derived principal components; and a polygenic score generated by REGENIE, which robustly adjusts for relatedness and population[12]. Association results from Geisinger Health System MyCode and the Mount Sinai BioMe Biobank were meta-analyzed using fixed-effects inverse variance weighting.

Protein abundance and RNA expression across cardiac cell types in human hearts

To evaluate protein abundance levels of *TTN*, *RPL3L*, *PKP2*, *CTNNA3*, *C10orf71*, and *KDM5B*, we used utilized mass spectrometry (MS)-based protein abundance measurements from human left and right atrial tissue of seven individuals from one of our previous studies[13]. Raw data were searched against the SwissProt human protein database containing canonical and isoform sequences using MaxQuant v1.5.3.19. ProteinsGroups.txt data were further processed and visualized using Python 3.7.1 and Seaborn 0.9.0. Reverse identifications, potential contaminants as well as proteins only identified by site were removed and LFQ protein intensities were extracted. One sample from the left atrium (H117-LA) showed a low number of protein identifications and a significantly lower overall protein intensity distribution and was thus removed from further analyses. Median protein intensity-based absolute quantification (iBAQ) values over all samples per atrium were calculated for each protein and visualized by means of a rank plot. *KDM5B* was not identified in the data set. Moreover, protein iBAQ values of *TTN*, *RPL3L*, *PKP2*, *CTNNA3*, and *C10orf71* of each biological replicate were extracted and visualized using a box plot.

Similarly, to evaluate in which cell types the proteins of interest are expressed in the human heart, we queried a publicly available single-nucleus RNA sequencing (snRNAseq) data set of 287,269 cells of the human heart published by Tucker et al.[14]. Cytoplasmic cardiomyocyte clusters were removed, the remaining clusters were combined and average RNA expression values per cell types were calculated as described by Tucker et al.[14]. The average expression values of *TTN*, *RPL3L*,

© 2024 Vad OB et al. *JAMA Cardiology.*

PKP2, *CTNNA3*, *C10orf71*, *KDM5B*, and *MYBPC3* were extracted and scaled per gene by dividing by the max expression value over all cell types. Data were processed and visualized using Python 3.7.1 and Seaborn 0.9.0. Results are illustrated in **eFigure 1**.

As the *C10orf71* had not previously been associated with cardiovascular phenotypes, we obtained tissue specific expression based on normalized consensus RNA-sequencing data from the Human Protein Atlas (Human Protein Atlas: www.proteinatlas.org)[15] and GTEx (www.gtexportal.org). The tissue specific RNA-expression of *C10orf71* was visualized using R. Results are illustrated in **eFigure 2**.

Risk of heart failure and cardiomyopathy

We assessed hazard ratios for incident AF, HF, and cardiomyopathy as separate outcomes. To ascertain temporal trends in incident disease, we considered each individual outcome and all-cause mortality as competing events. The models were adjusted for sex, age, BMI at inclusion, and hypertension, and IHD at inclusion. We considered $P<0.0056$ as statistically significant (3 genetic exposures x 3 independent outcomes).

Among individuals diagnosed with AF during follow-up, we assessed the hazard ratios for incident HF and cardiomyopathy based on carrier status of a rare pLOF variant. Individuals who developed HF or cardiomyopathy before AF were excluded. Hazard ratios were estimated for HF and cardiomyopathy as separate, competing events. The models were adjusted for sex, age at AF diagnosis, BMI, and hypertension or IHD at time of AF diagnosis.

Using a similar approach, we also estimated hazard ratios for incident AF in individuals diagnosed with either HF or cardiomyopathy during follow-up. Individuals that developed AF prior to HF/cardiomyopathy diagnosis were excluded from analyses. The models were adjusted for sex, age at HF/cardiomyopathy diagnosis, BMI, and hypertension or IHD at time of HF/cardiomyopathy diagnosis.

As a sensitivity analysis we evaluated the above models in a subset of unrelated individuals. In the subgroups of individuals with AF or HF/cardiomyopathy during follow-up, we applied a grace period and started follow-up 30 days after AF diagnosis."

© 2024 Vad OB et al. *JAMA Cardiology.*

eTable 1. Odds ratio for AF according to PRS and pLOF variants

Group	Odds ratio	Lower 95% CI	Upper 95% CI
0-20% PRS	Reference	-	-
20-40% PRS	1.37	1.31	1.43
40-60% PRS	1.62	1.55	1.69
60-80% PRS	2.01	1.93	2.10
80-100% PRS	2.96	2.84	3.09
0-20% PRS + pLOF variant	1.68	1.32	2.11
20-40% PRS + pLOF variant	2.62	2.13	3.21
40-60% PRS + pLOF variant	4.09	3.39	4.91
60-80% PRS + pLOF variant	4.34	3.63	5.15
80-100% PRS. + pLOF variant	7.08	6.03	8.28

CI, Confidence interval, pLOF variant, predicted loss-of-function variant in atrial fibrillation associated gene; PRS, Polygenic risk score for atrial fibrillation.

© 2024 Vad OB et al. *JAMA Cardiology.*

eTable 2. Odds ratio for AF according to PRS and pLOF variants (unrelated individuals)

Group	Odds ratio	Lower 95% CI	Upper 95% CI
0-20% PRS	Reference	-	-
20-40% PRS	1.38	1.31	1.45
40-60% PRS	1.65	1.57	1.73
60-80% PRS	2.03	1.94	2.13
80-100% PRS	3.01	2.88	3.15
0-20% PRS + pLOF variant	1.86	1.42	2.38
20-40% PRS + pLOF variant	2.61	2.07	3.25
40-60% PRS + pLOF variant	4.15	3.37	5.07
60-80% PRS + pLOF variant	4.59	3.78	5.53
80-100% PRS. + pLOF variant	7.72	6.48	9.16

CI, Confidence interval, pLOF variant, predicted loss-of-function variant in atrial fibrillation associated gene; PRS, Polygenic risk score for atrial fibrillation.

© 2024 Vad OB et al. *JAMA Cardiology.*

eTable 3. Variant carriers in study cohort for incident AF, HF and cardiomyopathy

Gene	Carriers, n (%)
TTN (PSI90)	1121 (0.28)
RPL3L	2972 (0.75)
PKP2	512 (0.13)
CTNNA3	221 (0.06)
KDM5B	223 (0.06)
**Variant carriers total**	**5032 (1.27)**

Carriers of rare pLOF variants in main study cohort after exclusion of individuals with prevalent AF, HF or cardiomyopathy. Fifteen individuals carried rare variants in two different genes. Variant carriers total denotes number of individuals with at least one pLOF variant.

© 2024 Vad OB et al. *JAMA Cardiology.*

eTable 4. Hazard ratios ratio for incident AF according to genetic risk and clinical risk factors

Covariate	Odds ratio	95% CI	P-value
0-20% PRS	Ref	-	-
20-40% PRS	1.31	[1.25;1.37]	<.001
40-60% PRS	1.54	[1.47;1.61]	<.001
60-80% PRS	1.84	[1.76;1.93]	<.001
80-100% PRS	2.53	[2.42;2.64]	<.001
0-20% PRS + pLOF variant	1.70	[1.33;2.17]	<.001
20-40% PRS + pLOF variant	2.16	[1.73;2.71]	<.001
40-60% PRS + pLOF variant	3.14	[2.57;3.83]	<.001
60-80% PRS + pLOF variant	3.59	[3.01;4.28]	<.001
80-100% PRS. + pLOF variant	4.78	[4.06;5.63]	<.001
Sex (male)	1.83	[1.78;1.88]	<.001
Age at inclusion (per year)	1.11	[1.11;1.11]	<.001
Obesity (BMI>30)	1.54	[1.50;1.59]	<.001
Hypertension	1.50	[1.46;1.54]	<.001
Ischemic Heart disease	1.65	[1.59;1.72]	<.001

BMI, Body-mass index, CI, Confidence interval, pLOF variant, predicted loss-of-function variant in atrial fibrillation associated gene; PRS, Polygenic risk score for atrial fibrillation.

© 2024 Vad OB et al. *JAMA Cardiology.*

eTable 5. Cumulative incidence of AF by age 80

Group	Crude cumulative incidence (%)	Lower 95% CI (%)	Upper 95% CI (%)
0-20% PRS	8.12	7.79	8.45
20-40% PRS	10.49	10.12	10.87
40-60% PRS	12.13	11.74	12.53
60-80% PRS	14.1	13.68	14.52
80-100% PRS	18.66	18.19	19.14
0-20% PRS + pLOF variant	12.48	9.18	15.77
20-40% PRS + pLOF variant	13.47	10.37	16.56
40-60% PRS + pLOF variant	22.9	18.19	27.62
60-80% PRS + pLOF variant	24.98	20.41	29.55
80-100% PRS. + pLOF variant	28.55	24.11	32.99

CI, Confidence interval, pLOF variant, predicted loss-of-function variant in atrial fibrillation associated gene; PRS, Polygenic risk score for atrial fibrillation.

© 2024 Vad OB et al. *JAMA Cardiology.*

eTable 6. Cumulative incidence of AF by age 70

Group	Crude cumulative incidence (%)	Lower 95% CI (%)	Upper 95% CI (%)
0-20% PRS	2.16	2.04	2.28
20-40% PRS	2.88	2.74	3.02
40-60% PRS	3.44	3.29	3.59
60-80% PRS	4.12	3.95	4.29
80-100% PRS	5.99	5.79	6.19
0-20% PRS + pLOF variant	3.18	1.94	4.42
20-40% PRS + pLOF variant	7.13	5.19	9.07
40-60% PRS + pLOF variant	6.56	4.67	8.45
60-80% PRS + pLOF variant	6.65	4.87	8.42
80-100% PRS. + pLOF variant	11.61	9.25	13.97

CI, Confidence interval, pLOF variant, predicted loss-of-function variant in atrial fibrillation associated gene; PRS, Polygenic risk score for atrial fibrillation.

© 2024 Vad OB et al. *JAMA Cardiology.*

eTable 7. Cumulative incidence of AF by age 60

Group	Crude cumulative incidence (%)	Lower 95% CI (%)	Upper 95% CI (%)
0-20% PRS	0.31	0.27	0.35
20-40% PRS	0.44	0.39	0.49
40-60% PRS	0.5	0.45	0.55
60-80% PRS	0.61	0.56	0.67
80-100% PRS	0.97	0.89	1.04
0-20% PRS + pLOF variant	0.29	0	0.62
20-40% PRS + pLOF variant	0.44	0.01	0.87
40-60% PRS + pLOF variant	1.23	0.51	1.95
60-80% PRS + pLOF variant	1.55	0.77	2.33
80-100% PRS. + pLOF variant	3.63	2.41	4.85

CI, Confidence interval, pLOF variant, predicted loss-of-function variant in atrial fibrillation associated gene; PRS, Polygenic risk score for atrial fibrillation.

© 2024 Vad OB et al. *JAMA Cardiology.*

eTable 8. Cumulative incidence of AF by age 80 (unrelated individuals)

Group	Crude cumulative incidence (%)	Lower 95% CI (%)	Upper 95% CI (%)
0-20% PRS	7.87	7.51	8.24
20-40% PRS	10.47	10.06	10.88
40-60% PRS	12.14	11.69	12.58
60-80% PRS	14.00	13.54	14.47
80-100% PRS	18.72	18.19	19.25
0-20% PRS + pLOF variant	12.92	9.46	16.38
20-40% PRS + pLOF variant	12.44	9.25	15.63
40-60% PRS + pLOF variant	22.03	17.09	26.98
60-80% PRS + pLOF variant	26.18	21.02	31.34
80-100% PRS. + pLOF variant	29.59	24.59	34.59

CI, Confidence interval, pLOF variant, predicted loss-of-function variant in atrial fibrillation associated gene; PRS, Polygenic risk score for atrial fibrillation.

© 2024 Vad OB et al. *JAMA Cardiology.*

eTable 9. Cumulative incidence of AF by age 80 (excluding TTN pLOF variants)

Group	Crude cumulative incidence (%)	Lower 95% CI (%)	Upper 95% CI (%)
0-20% PRS	8.15	7.81	8.48
20-40% PRS	10.53	10.16	10.9
40-60% PRS	12.19	11.8	12.59
60-80% PRS	14.16	13.74	14.58
80-100% PRS	18.72	18.25	19.19
0-20% PRS + pLOF variant	11.24	7.78	14.71
20-40% PRS + pLOF variant	10.85	7.92	13.79
40-60% PRS + pLOF variant	19.05	14.54	23.55
60-80% PRS + pLOF variant	22.75	17.64	27.86
80-100% PRS. + pLOF variant	25.54	20.66	30.43

CI, Confidence interval, pLOF variant, predicted loss-of-function variant in atrial fibrillation associated gene; PRS, Polygenic risk score for atrial fibrillation.

© 2024 Vad OB et al. *JAMA Cardiology.*

© 2024 Vad OB et al. *JAMA Cardiology*.

eFigure 1. Flowchart of study design

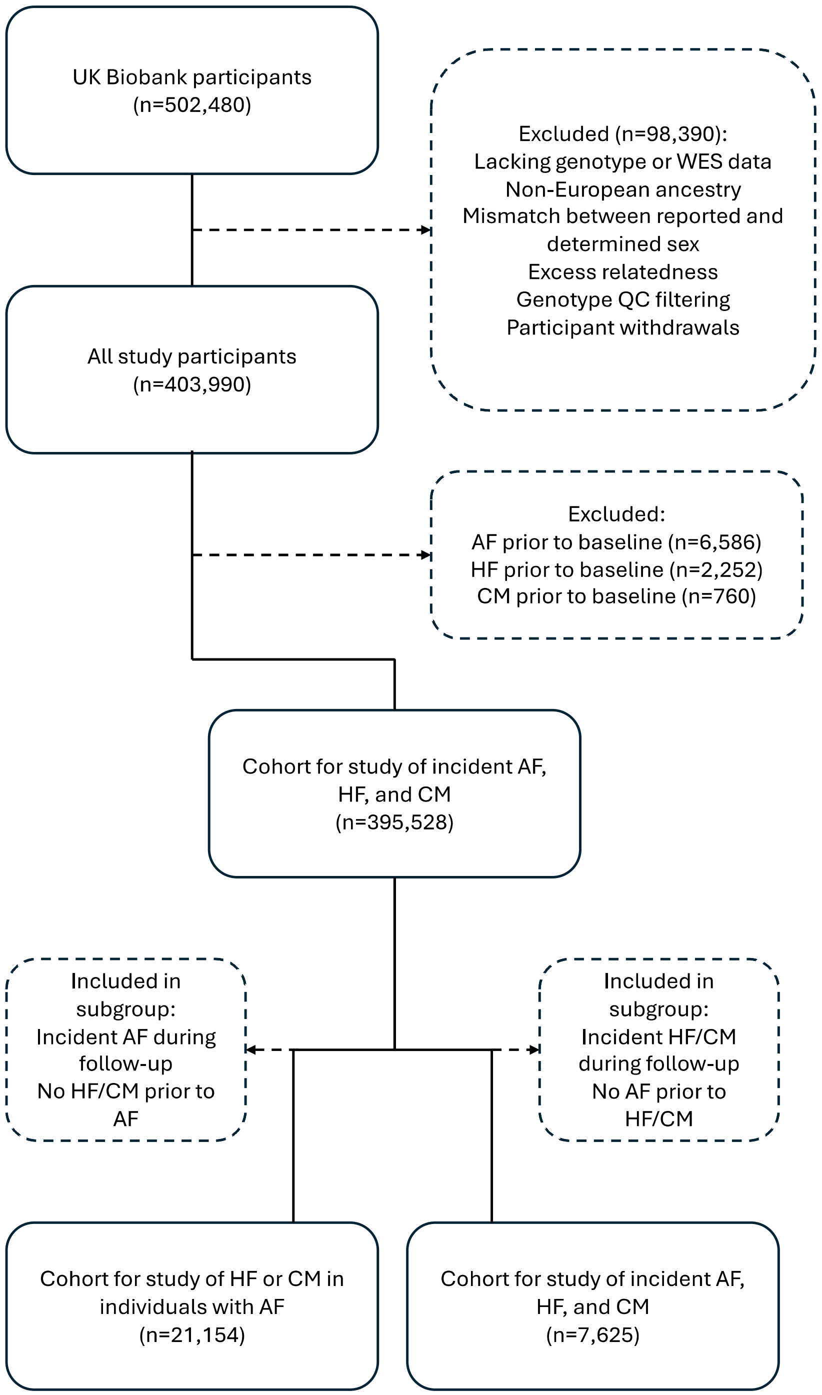

[illegible] control, WES, whole-exome sequencing.

© 2024 Vad OB et al. *JAMA Cardiology.*

eFigure 2. Manhattan plot of gene-based test for rare pLOF variants

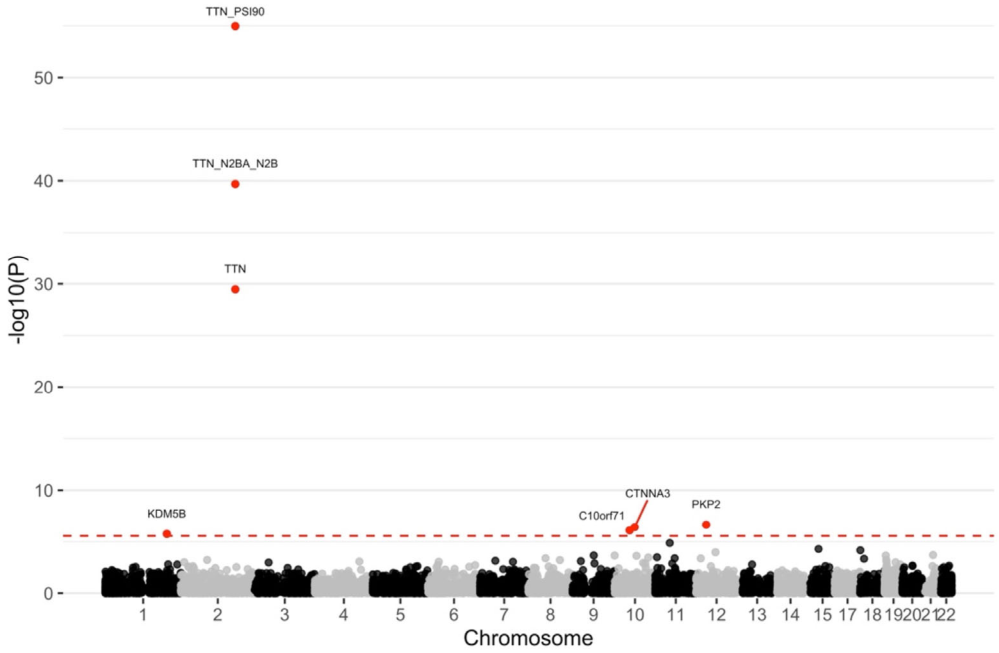

X-axis denotes chromosomal position of the gene. Y-axis denotes -log10 of the P-value for the genetic associations with AF. Significant genes are labeled and colored in red.

eFigure 3. Quantile-Quantile plot of gene-based test for rare pLOF variants

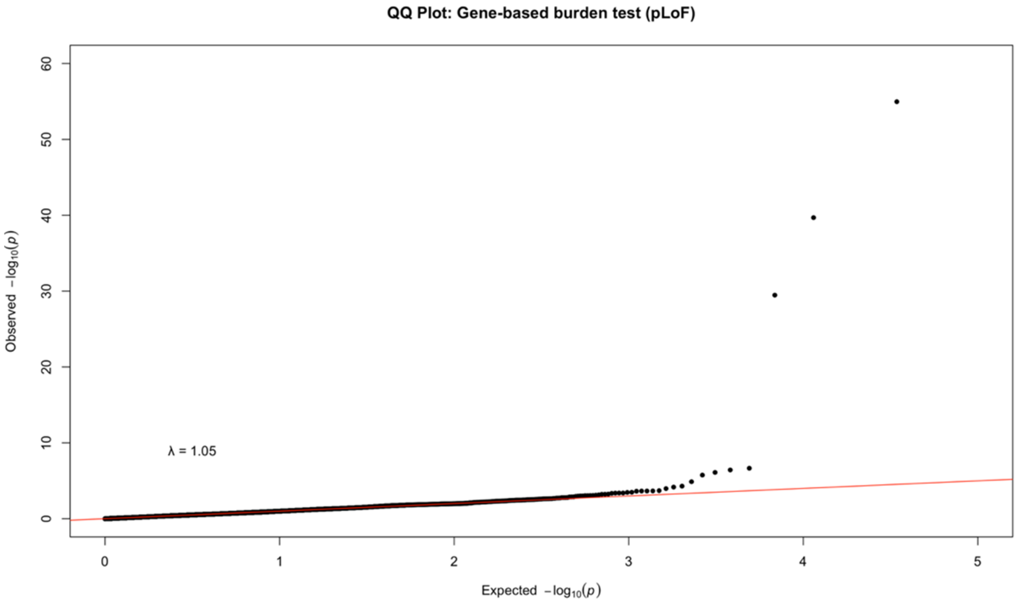

X-axis denotes expected -log10 P-value, while Y-axis denotes the observed -log10 P-values. The lambda value (**λ)** indicates a measure of genomic inflation in the dataset.

© 2024 Vad OB et al. *JAMA Cardiology.*

eFigure 4. Cardiac expression of AF associated genes

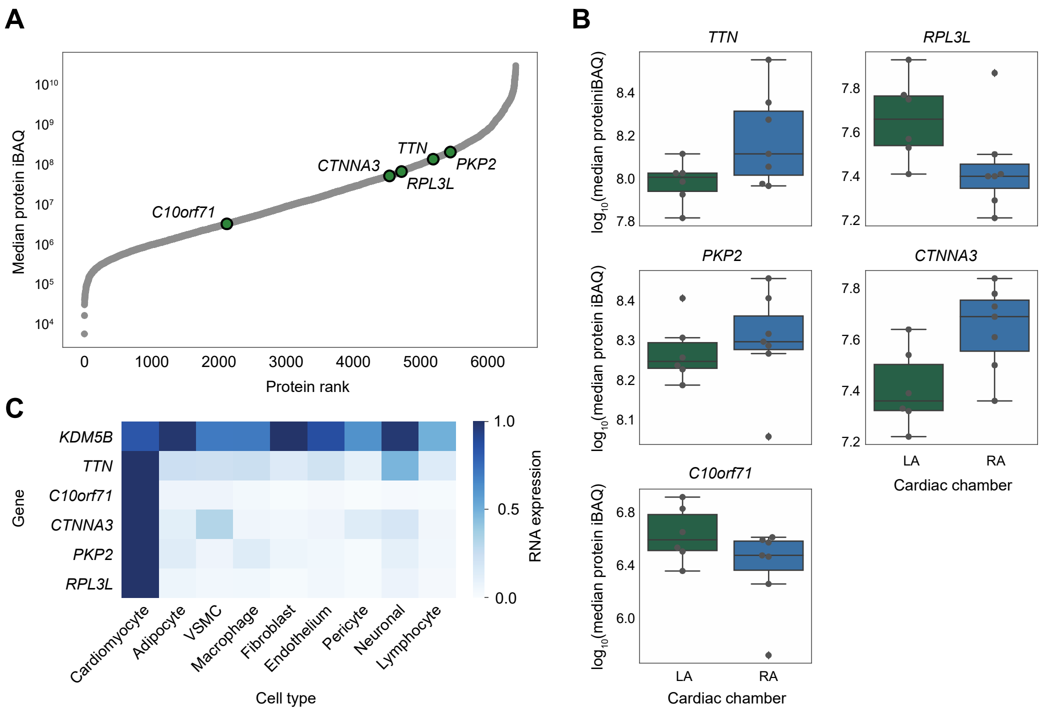

Suppl. Figure 1A) relative abundance of protein products of the AF-associated genes identified in the study. Suppl. Figure 1B) relative abundance of protein products in left atria (LA) and right atria (RA) respectively. The product of *KDM5B* was not identified in the proteomics dataset. Suppl. Figure 1C) relative RNA expression across cell types, based on single-cell RNA expression data.

© 2024 Vad OB et al. *JAMA Cardiology.*

eFigure 5. Tissue-specific RNA expression of *C10orf71*

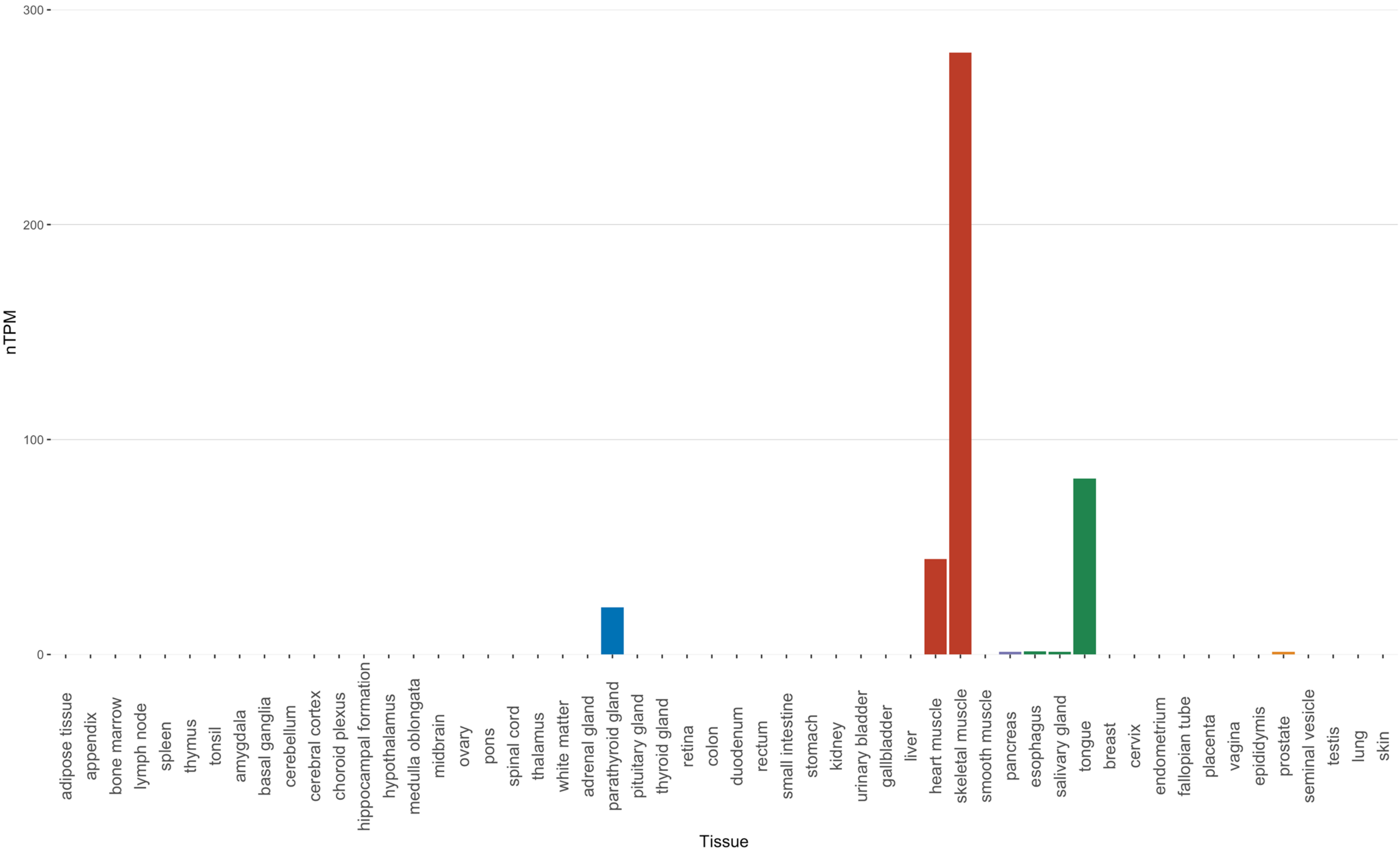

© 2024 Vad OB et al. *JAMA Cardiology*.

eFigure 6. Results from gene-based association test with AF in independent replication cohort

Exposure	N (Ref/Het/Alt)	OR (95% CI)	P-value
TTN, pLoF, MAF < 1%	case:17544/366/0 control:146944/2401/3	1.67 (1.45, 1.92)	3.52e-13
RPL3L, pLoF, MAF < 1%	case:17816/94/0 control:148772/575/1	1.34 (1.03, 1.74)	0.027
PKP2, pLoF, MAF < 1%	case:17887/23/0 control:149198/150/0	1.87 (1.04, 3.37)	0.036
CTNNA3, pLoF, MAF < 1%	case:17875/35/0 control:149143/205/0	1.59 (1.02, 2.50)	0.042
C10orf71, pLoF, MAF < 1%	case:17858/52/0 control:149057/291/0	1.28 (0.88, 1.84)	0.193
KDM5B, pLoF, MAF < 1%	case:17874/36/0 control:149153/195/0	2.13 (1.37, 3.31)	0.001

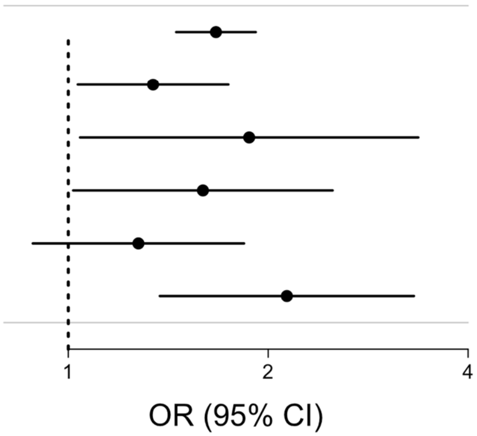

Forest plot of odds ratios (OR) and confidence intervals of AF associated genes in external cohort. MAF, minor allele frequence (alternate allele), Alt, homozygous for alternate allele, Ref, heterozygous for alternate allele, Ref, homozygous for reference allele allele, OR, odds ratio, CI, confidence interval.

© 2024 Vad OB et al. *JAMA Cardiology*.

eFigure 7. Ten-year risk of AF (unrelated individuals)

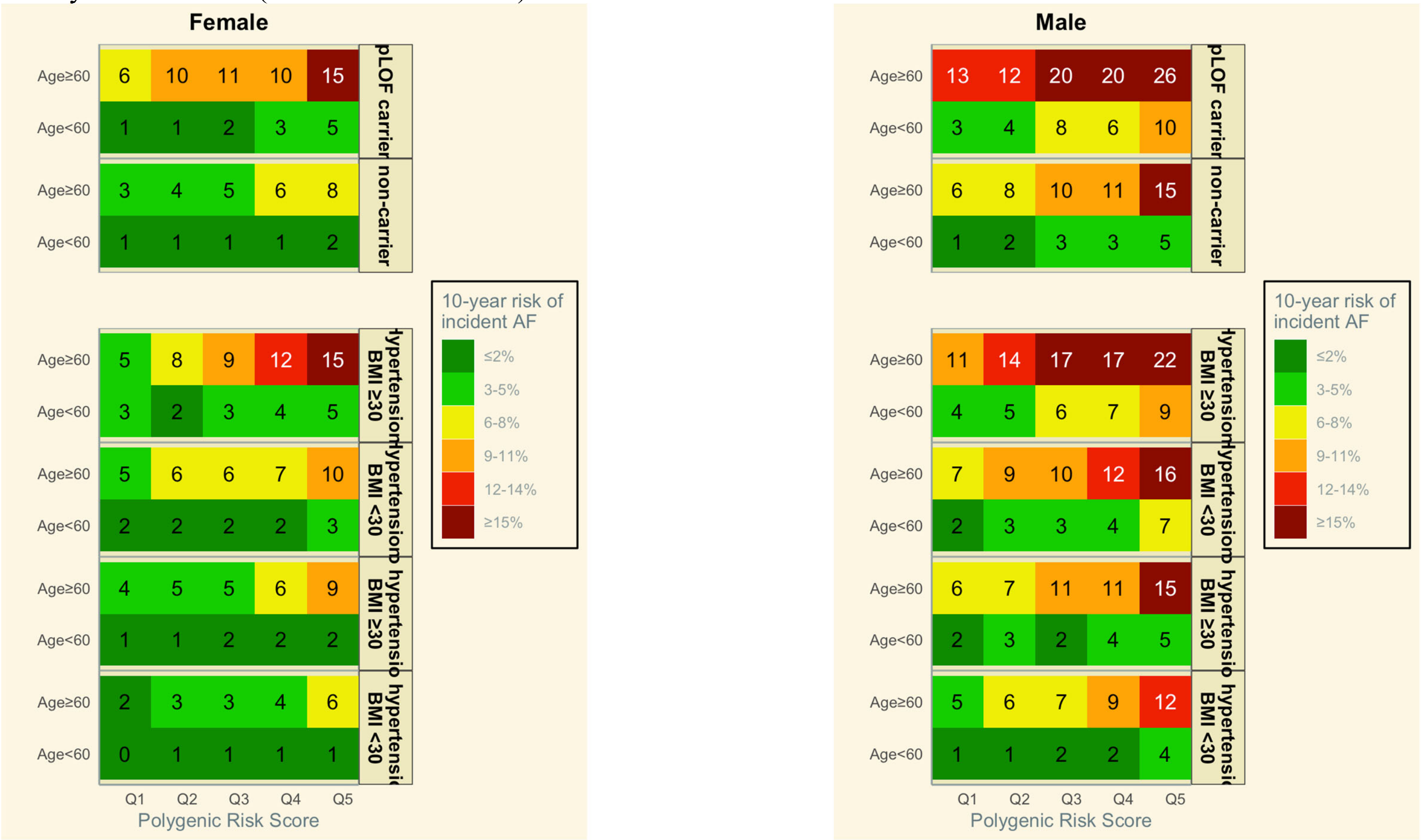

© 2024 Vad OB et al. *JAMA Cardiology.*

eFigure 8. Ten-year risk of AF (excluding TTN pLOF variants)

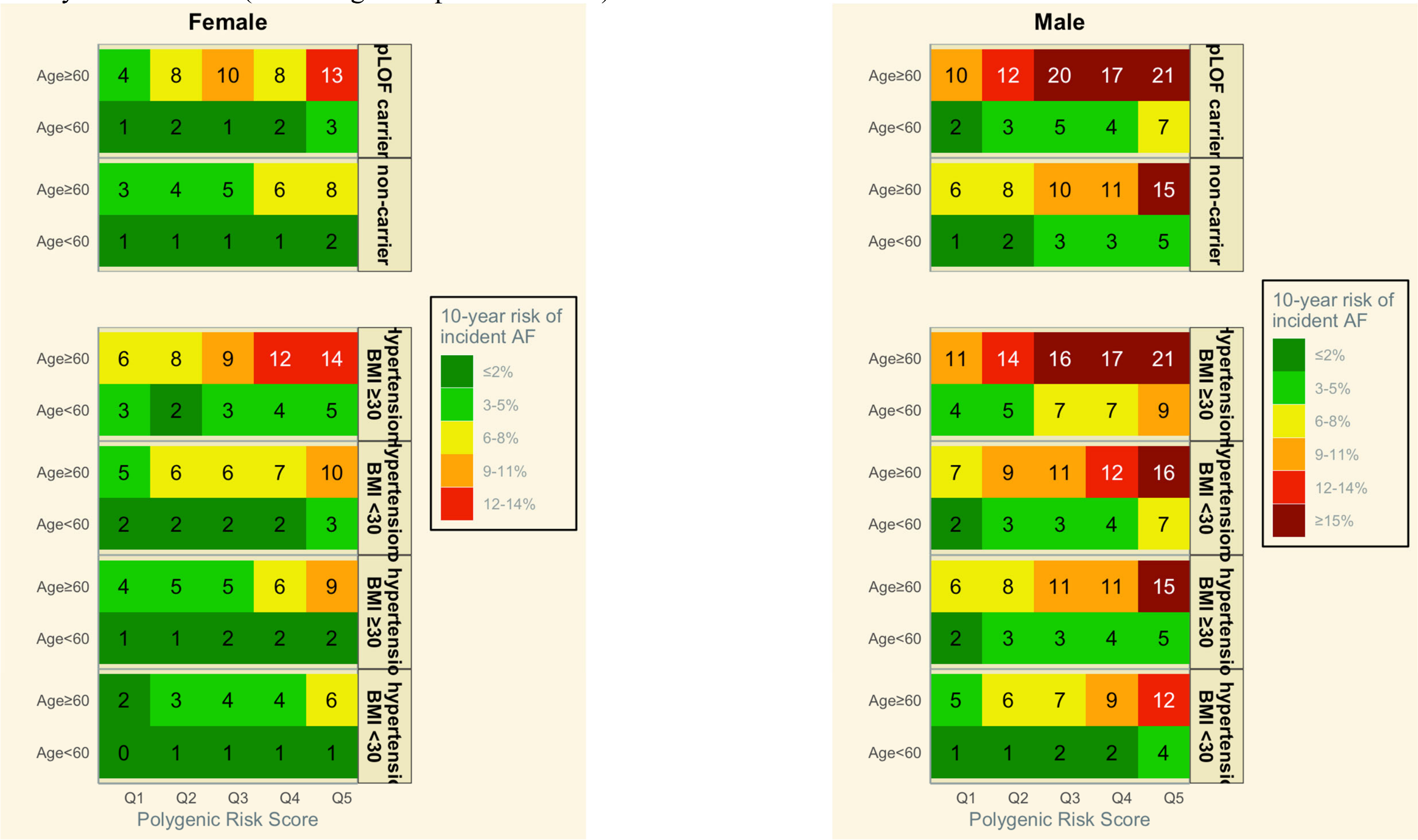

© 2024 Vad OB et al. *JAMA Cardiology*.

eFigure 9. Forest plot of hazard ratios for AF, cardiomyopathy, and HF (unrelaed individuals)

Group	Outcome	HR (95% CI)	P-value
Full cohort (unrelated)		1.92 (1.74, 2.12)	<0.001
(N=325,872)	Atrial Fibrillation	1.66 (1.48, 1.86)	<0.001
		1.46 (1.44, 1.48)	<0.001
		2.56 (1.71, 3.85)	<0.001
	Cardiomyopathy	1.45 (0.80, 2.63)	0.22
		0.97 (0.90, 1.05)	0.452
		1.58 (1.29, 1.93)	<0.001
	Heart Failure	1.18 (0.91, 1.52)	0.207
		1.04 (1.01, 1.07)	0.016
Diagnosed with AF (unrelated)		2.99 (1.82, 4.89)	<0.001
(N=17,235)	Cardiomyopathy	1.21 (0.50, 2.94)	0.672
		1.05 (0.92, 1.20)	0.459
		1.20 (0.93, 1.56)	0.168
	Heart Failure	0.80 (0.55, 1.15)	0.226
		1.01 (0.96, 1.05)	0.788
Diagnosed with CM/HF (unrelated)		1.28 (0.88, 1.85)	0.199
(N=6,184)	Atrial Fibrillation	1.73 (1.03, 2.89)	0.037
		1.03 (0.96, 1.11)	0.39

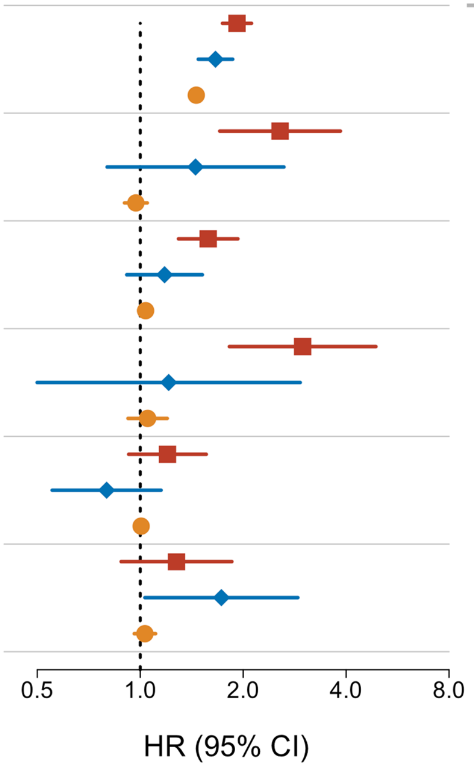

© 2024 Vad OB et al. *JAMA Cardiology*.

eFigure 10. Forest plot of hazard ratios for AF, cardiomyopathy, and HF (30-day grace period)

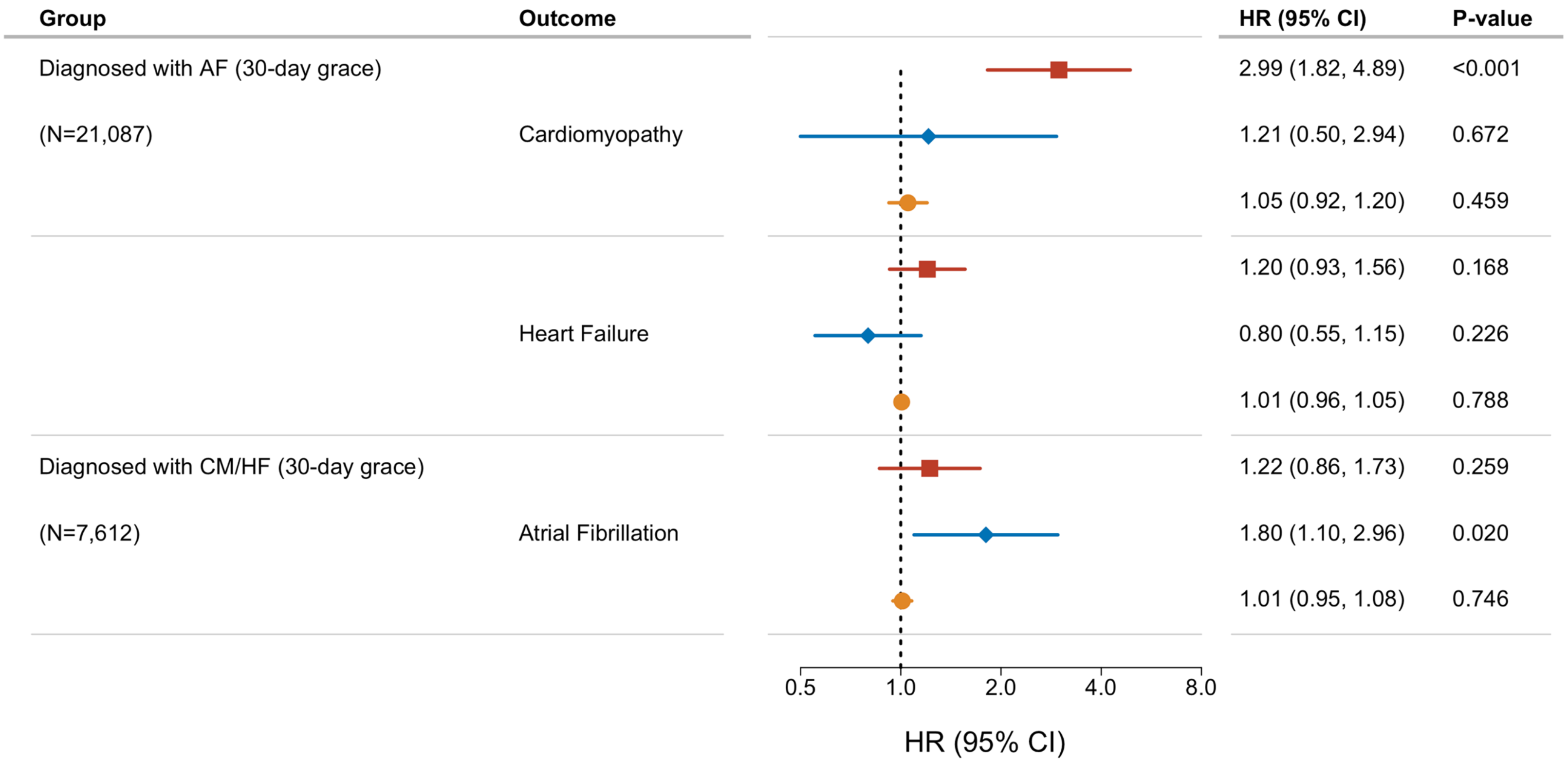

© 2024 Vad OB et al. *JAMA Cardiology.*

References

1. Sherry ST, Ward MH, Kholodov M, et al. dbSNP: the NCBI database of genetic variation. *Nucleic Acids Res*. 2001;29(1):308-311.
2. Cingolani P, Platts A, Wang LL, et al. A program for annotating and predicting the effects of single nucleotide polymorphisms, SnpEff: SNPs in the genome of Drosophila melanogaster strain w1118; iso-2; iso-3. *Fly (Austin)*. 2012;6(2):80-92. doi:10.4161/fly.19695
3. Jaganathan K, Panagiotopoulou SK, McRae JF, et al. Predicting Splicing from Primary Sequence with Deep Learning. *Cell*. 2019;176(3):535-548.e24. doi:10.1016/j.cell.2018.12.015
4. Wu MC, Lee S, Cai T, Li Y, Boehnke M, Lin X. Rare-Variant Association Testing for Sequencing Data with the Sequence Kernel Association Test. *Am J Hum Genet*. 2011;89(1):82-93. doi:10.1016/j.ajhg.2011.05.029
5. Liu Y, Chen S, Li Z, Morrison AC, Boerwinkle E, Lin X. ACAT: A Fast and Powerful p Value Combination Method for Rare-Variant Analysis in Sequencing Studies. *Am J Hum Genet*. 2019;104(3):410-421. doi:10.1016/j.ajhg.2019.01.002
6. Verweij N, Haas ME, Nielsen JB, et al. Germline Mutations in CIDEB and Protection against Liver Disease. *New England Journal of Medicine*. 2022;387(4):332-344. doi:10.1056/NEJMoa2117872
7. Taliun D, Harris DN, Kessler MD, et al. Sequencing of 53,831 diverse genomes from the NHLBI TOPMed Program. *Nature*. 2021;590(7845):290-299. doi:10.1038/s41586-021-03205-y
8. Das S, Forer L, Schönherr S, et al. Next-generation genotype imputation service and methods. *Nat Genet*. 2016;48(10):1284-1287. doi:10.1038/ng.3656
9. Fuchsberger C, Abecasis GR, Hinds DA. minimac2: faster genotype imputation. *Bioinformatics*. 2015;31(5):782-784. doi:10.1093/bioinformatics/btu704
10. Akbari P, Gilani A, Sosina O, et al. Sequencing of 640,000 exomes identifies GPR75 variants associated with protection from obesity. *Science*. 2021;373(6550):eabf8683. doi:10.1126/science.abf8683
11. Akbari P, Sosina OA, Bovijn J, et al. Multiancestry exome sequencing reveals INHBE mutations associated with favorable fat distribution and protection from diabetes. *Nat Commun*. 2022;13(1):4844. doi:10.1038/s41467-022-32398-7
12. Mbatchou J, Barnard L, Backman J, et al. Computationally efficient whole-genome regression for quantitative and binary traits. *Nat Genet*. 2021;53(7):1097-1103. doi:10.1038/s41588-021-00870-7
13. Linscheid N, Poulsen PC, Pedersen ID, et al. Quantitative Proteomics of Human Heart Samples Collected In Vivo Reveal the Remodeled Protein Landscape of Dilated Left Atrium Without Atrial Fibrillation. *Mol Cell Proteomics*. 2020;19(7):1132-1144. doi:10.1074/mcp.RA119.001878
14. Tucker NR, Chaffin M, Fleming SJ, et al. Transcriptional and Cellular Diversity of the Human Heart. *Circulation*. 2020;142(5):466-482. doi:10.1161/CIRCULATIONAHA.119.045401
15. Uhlén M, Fagerberg L, Hallström BM, et al. Proteomics. Tissue-based map of the human proteome. *Science*. 2015;347(6220):1260419. doi:10.1126/science.1260419

© 2024 Vad OB et al. *JAMA Cardiology.*
