# Supplementary material for: Rare and Common Genetic Variation Underlying Atrial Fibrillation Risk
Source: JAMA Cardiol. 2024 Jun 26;9(8):732–40. doi: 10.1001/jamacardio.2024.1528 (PMC11209175; doi:10.1001/jamacardio.2024.1528)
Supplement: Supplement 3. — Geisinger MyCode Community Health Initiative and Regeneron Genetics Center (RGC) Research Team members [file jamacardiol-e241528-s003.pdf]

Supplemental Online Content: Nonauthor Collaborators

*Indicates required information. Only first name, last name, and suffix will appear in PubMed.

***Group Name(s): The Geisinger MyCode Community Health Initiative, Regeneron Genetics Center (RGC) Research Team**									
***First Name and Middle Initial(s)**	***Last Name**	***Suffix (eg, Jr, III)**	Academic Degrees	Institution	Location (city, state/province, country)	Role or Contribution, eg, chair, principal investigator	Group (if more than 1 Group listed in the byline) and/or Subgroup (eg, Steering Committee)		
Aris	Baras			Regeneron Genetics Center	Tarrytown, NY, USA	RGC Management & Leadership Team	Regeneron Genetics Center		
Gonçalo	Abecasis			Regeneron Genetics Center	Tarrytown, NY, USA	RGC Management & Leadership Team	Regeneron Genetics Center		
Adolfo	Ferrando			Regeneron Genetics Center	Tarrytown, NY, USA	RGC Management & Leadership Team	Regeneron Genetics Center		
Michael	Cantor			Regeneron Genetics Center	Tarrytown, NY, USA	RGC Management & Leadership Team	Regeneron Genetics Center		
Giovanni	Coppola			Regeneron Genetics Center	Tarrytown, NY, USA	RGC Management & Leadership Team	Regeneron Genetics Center		
Andrew	Deubler			Regeneron Genetics Center	Tarrytown, NY, USA	RGC Management & Leadership Team	Regeneron Genetics Center		
Aris	Economides			Regeneron Genetics Center	Tarrytown, NY, USA	RGC Management & Leadership Team	Regeneron Genetics Center		
Luca A	Lotta			Regeneron Genetics Center	Tarrytown, NY, USA	RGC Management & Leadership Team	Regeneron Genetics Center		
John D	Overton			Regeneron Genetics Center	Tarrytown, NY, USA	RGC Management & Leadership Team	Regeneron Genetics Center		
Jeffrey G	Reid			Regeneron Genetics Center	Tarrytown, NY, USA	RGC Management & Leadership Team	Regeneron Genetics Center		
Alan	Shuldiner			Regeneron Genetics Center	Tarrytown, NY, USA	RGC Management & Leadership Team	Regeneron Genetics Center		
Katherine	Siminovitch			Regeneron Genetics Center	Tarrytown, NY, USA	RGC Management & Leadership Team	Regeneron Genetics Center		
Jason	Portnoy			Regeneron Genetics Center	Tarrytown, NY, USA	RGC Management & Leadership Team	Regeneron Genetics Center		
Marcus B	Jones			Regeneron Genetics Center	Tarrytown, NY, USA	RGC Management & Leadership Team	Regeneron Genetics Center		
Lyndon	Mitnaul			Regeneron Genetics Center	Tarrytown, NY, USA	RGC Management & Leadership Team	Regeneron Genetics Center		
Alison	Fenney			Regeneron Genetics Center	Tarrytown, NY, USA	RGC Management & Leadership Team	Regeneron Genetics Center		
Jonathan	Marchini			Regeneron Genetics Center	Tarrytown, NY, USA	RGC Management & Leadership Team	Regeneron Genetics Center		
Manuel AR	Ferreira			Regeneron Genetics Center	Tarrytown, NY, USA	RGC Management & Leadership Team	Regeneron Genetics Center		
Maya	Ghoussaini			Regeneron Genetics Center	Tarrytown, NY, USA	RGC Management & Leadership Team	Regeneron Genetics Center		
Mona	Nafde			Regeneron Genetics Center	Tarrytown, NY, USA	RGC Management & Leadership Team	Regeneron Genetics Center		
William	Salerno			Regeneron Genetics Center	Tarrytown, NY, USA	RGC Management & Leadership Team	Regeneron Genetics Center		
John D	Overton			Regeneron Genetics Center	Tarrytown, NY, USA	Sequencing & Lab Operations	Regeneron Genetics Center		
Christina	Beechert			Regeneron Genetics Center	Tarrytown, NY, USA	Sequencing & Lab Operations	Regeneron Genetics Center		
Erin D	Brian			Regeneron Genetics Center	Tarrytown, NY, USA	Sequencing & Lab Operations	Regeneron Genetics Center		
Laura M	Cremona			Regeneron Genetics Center	Tarrytown, NY, USA	Sequencing & Lab Operations	Regeneron Genetics Center		
Hang	Du			Regeneron Genetics Center	Tarrytown, NY, USA	Sequencing & Lab Operations	Regeneron Genetics Center		
Caitlin	Forsythe			Regeneron Genetics Center	Tarrytown, NY, USA	Sequencing & Lab Operations	Regeneron Genetics Center		
Zhenhua	Gu			Regeneron Genetics Center	Tarrytown, NY, USA	Sequencing & Lab Operations	Regeneron Genetics Center		
Kristy	Guevara			Regeneron Genetics Center	Tarrytown, NY, USA	Sequencing & Lab Operations	Regeneron Genetics Center		
Michael	Lattari			Regeneron Genetics Center	Tarrytown, NY, USA	Sequencing & Lab Operations	Regeneron Genetics Center		
Alexander	Lopez			Regeneron Genetics Center	Tarrytown, NY, USA	Sequencing & Lab Operations	Regeneron Genetics Center		
Kia	Manoochehri			Regeneron Genetics Center	Tarrytown, NY, USA	Sequencing & Lab Operations	Regeneron Genetics Center		
Prathyusha	Challa			Regeneron Genetics Center	Tarrytown, NY, USA	Sequencing & Lab Operations	Regeneron Genetics Center		
Manasi	Pradhan			Regeneron Genetics Center	Tarrytown, NY, USA	Sequencing & Lab Operations	Regeneron Genetics Center		
Raymond	Reynoso			Regeneron Genetics Center	Tarrytown, NY, USA	Sequencing & Lab Operations	Regeneron Genetics Center		
Ricardo	Schiavo			Regeneron Genetics Center	Tarrytown, NY, USA	Sequencing & Lab Operations	Regeneron Genetics Center		
Maria S	Padilla			Regeneron Genetics Center	Tarrytown, NY, USA	Sequencing & Lab Operations	Regeneron Genetics Center		
Chenggu	Wang			Regeneron Genetics Center	Tarrytown, NY, USA	Sequencing & Lab Operations	Regeneron Genetics Center		
Sarah E	Wolf			Regeneron Genetics Center	Tarrytown, NY, USA	Sequencing & Lab Operations	Regeneron Genetics Center		
Michael	Cantor			Regeneron Genetics Center	Tarrytown, NY, USA	Clinical Informatics	Regeneron Genetics Center		

Supplemental Online Content: Nonauthor Collaborators

*Indicates required information. Only first name, last name, and suffix will appear in PubMed.

*First Name and Middle Initial(s)	*Last Name	*Suffix (eg, Jr, III)	Academic Degrees	Institution	Location (city, state/province, country)	Role or Contribution, eg, chair, principal investigator	Group (if more than 1 Group listed in the byline) and/or Subgroup (eg, Steering Committee)		
Amelia	Averitt			Regeneron Genetics Center	Tarrytown, NY, USA	Clinical Informatics	Regeneron Genetics Center		
Nilanjana	Banerjee			Regeneron Genetics Center	Tarrytown, NY, USA	Clinical Informatics	Regeneron Genetics Center		
Dadong	Li			Regeneron Genetics Center	Tarrytown, NY, USA	Clinical Informatics	Regeneron Genetics Center		
Sameer	Malhotra			Regeneron Genetics Center	Tarrytown, NY, USA	Clinical Informatics	Regeneron Genetics Center		
Justin	Mower			Regeneron Genetics Center	Tarrytown, NY, USA	Clinical Informatics	Regeneron Genetics Center		
Mudasar	Sarwar			Regeneron Genetics Center	Tarrytown, NY, USA	Clinical Informatics	Regeneron Genetics Center		
Deepika	Sharma			Regeneron Genetics Center	Tarrytown, NY, USA	Clinical Informatics	Regeneron Genetics Center		
Jeffrey C	Staples			Regeneron Genetics Center	Tarrytown, NY, USA	Clinical Informatics	Regeneron Genetics Center		
Sean	Yu			Regeneron Genetics Center	Tarrytown, NY, USA	Clinical Informatics	Regeneron Genetics Center		
Aaron	Zhang			Regeneron Genetics Center	Tarrytown, NY, USA	Clinical Informatics	Regeneron Genetics Center		
Muhammad	Aqeel			Regeneron Genetics Center	Tarrytown, NY, USA	Clinical Informatics	Regeneron Genetics Center		
Jeffrey G	Reid			Regeneron Genetics Center	Tarrytown, NY, USA	Genome Informatics & Data Engineering	Regeneron Genetics Center		
Mona	Nafde			Regeneron Genetics Center	Tarrytown, NY, USA	Genome Informatics & Data Engineering	Regeneron Genetics Center		
George	Mitra			Regeneron Genetics Center	Tarrytown, NY, USA	Genome Informatics & Data Engineering	Regeneron Genetics Center		
Sujit	Gokhale			Regeneron Genetics Center	Tarrytown, NY, USA	Genome Informatics & Data Engineering	Regeneron Genetics Center		
Andrew	Bunyea			Regeneron Genetics Center	Tarrytown, NY, USA	Genome Informatics & Data Engineering	Regeneron Genetics Center		
Krishna P	Punuru			Regeneron Genetics Center	Tarrytown, NY, USA	Genome Informatics & Data Engineering	Regeneron Genetics Center		
Sanjay	Sreeram			Regeneron Genetics Center	Tarrytown, NY, USA	Genome Informatics & Data Engineering	Regeneron Genetics Center		
Gisu	Eom			Regeneron Genetics Center	Tarrytown, NY, USA	Genome Informatics & Data Engineering	Regeneron Genetics Center		
Sujit	Gokhale			Regeneron Genetics Center	Tarrytown, NY, USA	Genome Informatics & Data Engineering	Regeneron Genetics Center		
Benjamin	Sultan			Regeneron Genetics Center	Tarrytown, NY, USA	Genome Informatics & Data Engineering	Regeneron Genetics Center		
Rouel	Lanche			Regeneron Genetics Center	Tarrytown, NY, USA	Genome Informatics & Data Engineering	Regeneron Genetics Center		
Vrushali	Mahajan			Regeneron Genetics Center	Tarrytown, NY, USA	Genome Informatics & Data Engineering	Regeneron Genetics Center		
Eliot	Austin			Regeneron Genetics Center	Tarrytown, NY, USA	Genome Informatics & Data Engineering	Regeneron Genetics Center		
Sean	O'Keeffe			Regeneron Genetics Center	Tarrytown, NY, USA	Genome Informatics & Data Engineering	Regeneron Genetics Center		
Razvan	Panea			Regeneron Genetics Center	Tarrytown, NY, USA	Genome Informatics & Data Engineering	Regeneron Genetics Center		
Tommy	Polanco			Regeneron Genetics Center	Tarrytown, NY, USA	Genome Informatics & Data Engineering	Regeneron Genetics Center		
Ayesha	Rasool			Regeneron Genetics Center	Tarrytown, NY, USA	Genome Informatics & Data Engineering	Regeneron Genetics Center		
William	Salerno			Regeneron Genetics Center	Tarrytown, NY, USA	Genome Informatics & Data Engineering	Regeneron Genetics Center		
Xiaodong	Bai			Regeneron Genetics Center	Tarrytown, NY, USA	Genome Informatics & Data Engineering	Regeneron Genetics Center		
Lance	Zhang			Regeneron Genetics Center	Tarrytown, NY, USA	Genome Informatics & Data Engineering	Regeneron Genetics Center		
Boris	Boutkov			Regeneron Genetics Center	Tarrytown, NY, USA	Genome Informatics & Data Engineering	Regeneron Genetics Center		
Evan	Edelstein			Regeneron Genetics Center	Tarrytown, NY, USA	Genome Informatics & Data Engineering	Regeneron Genetics Center		
Alexander	Gorovits			Regeneron Genetics Center	Tarrytown, NY, USA	Genome Informatics & Data Engineering	Regeneron Genetics Center		
Ju	Guan			Regeneron Genetics Center	Tarrytown, NY, USA	Genome Informatics & Data Engineering	Regeneron Genetics Center		
Lukas	Habegger			Regeneron Genetics Center	Tarrytown, NY, USA	Genome Informatics & Data Engineering	Regeneron Genetics Center		
Alicia	Hawes			Regeneron Genetics Center	Tarrytown, NY, USA	Genome Informatics & Data Engineering	Regeneron Genetics Center		
Olga	Krasheninina			Regeneron Genetics Center	Tarrytown, NY, USA	Genome Informatics & Data Engineering	Regeneron Genetics Center		
Samantha	Zarate			Regeneron Genetics Center	Tarrytown, NY, USA	Genome Informatics & Data Engineering	Regeneron Genetics Center		
Adam J	Mansfield			Regeneron Genetics Center	Tarrytown, NY, USA	Genome Informatics & Data Engineering	Regeneron Genetics Center		
Evan K	Maxwell			Regeneron Genetics Center	Tarrytown, NY, USA	Genome Informatics & Data Engineering	Regeneron Genetics Center		
Suganthi	Balasubramanian			Regeneron Genetics Center	Tarrytown, NY, USA	Genome Informatics & Data Engineering	Regeneron Genetics Center		

*Indicates required information. Only first name, last name, and suffix will appear in PubMed.

*First Name and Middle Initial(s)	*Last Name	*Suffix (eg, Jr, III)	Academic Degrees	Institution	Location (city, state/province, country)	Role or Contribution, eg, chair, principal investigator	Group (if more than 1 Group listed in the byline) and/or Subgroup (eg, Steering Committee)		
Suying	Bao			Regeneron Genetics Center	Tarrytown, NY, USA	Genome Informatics & Data Engineering	Regeneron Genetics Center		
Kathie	Sun			Regeneron Genetics Center	Tarrytown, NY, USA	Genome Informatics & Data Engineering	Regeneron Genetics Center		
Chuanyi	Zhang			Regeneron Genetics Center	Tarrytown, NY, USA	Genome Informatics & Data Engineering	Regeneron Genetics Center		
Vikhna Raj Kumar	Karuppaiya			Regeneron Genetics Center	Tarrytown, NY, USA	Genome Informatics & Data Engineering	Regeneron Genetics Center		
Gonçalo	Abecasis			Regeneron Genetics Center	Tarrytown, NY, USA	Analytical Genetics and Data Science	Regeneron Genetics Center		
Manuel AR	Ferreira			Regeneron Genetics Center	Tarrytown, NY, USA	Analytical Genetics and Data Science	Regeneron Genetics Center		
Joshua	Backman			Regeneron Genetics Center	Tarrytown, NY, USA	Analytical Genetics and Data Science	Regeneron Genetics Center		
Kathy	Burch			Regeneron Genetics Center	Tarrytown, NY, USA	Analytical Genetics and Data Science	Regeneron Genetics Center		
Adrian	Campos			Regeneron Genetics Center	Tarrytown, NY, USA	Analytical Genetics and Data Science	Regeneron Genetics Center		
Lei	Chen			Regeneron Genetics Center	Tarrytown, NY, USA	Analytical Genetics and Data Science	Regeneron Genetics Center		
Sam	Choi			Regeneron Genetics Center	Tarrytown, NY, USA	Analytical Genetics and Data Science	Regeneron Genetics Center		
Amy	Damask			Regeneron Genetics Center	Tarrytown, NY, USA	Analytical Genetics and Data Science	Regeneron Genetics Center		
Liron	Ganel			Regeneron Genetics Center	Tarrytown, NY, USA	Analytical Genetics and Data Science	Regeneron Genetics Center		
Sheila	Gaynor			Regeneron Genetics Center	Tarrytown, NY, USA	Analytical Genetics and Data Science	Regeneron Genetics Center		
Benjamin	Geraghty			Regeneron Genetics Center	Tarrytown, NY, USA	Analytical Genetics and Data Science	Regeneron Genetics Center		
Arkopravo	Ghosh			Regeneron Genetics Center	Tarrytown, NY, USA	Analytical Genetics and Data Science	Regeneron Genetics Center		
Salvador R	Martinez			Regeneron Genetics Center	Tarrytown, NY, USA	Analytical Genetics and Data Science	Regeneron Genetics Center		
Christopher	Gillies			Regeneron Genetics Center	Tarrytown, NY, USA	Analytical Genetics and Data Science	Regeneron Genetics Center		
Lauren	Gurski			Regeneron Genetics Center	Tarrytown, NY, USA	Analytical Genetics and Data Science	Regeneron Genetics Center		
Joseph	Herman			Regeneron Genetics Center	Tarrytown, NY, USA	Analytical Genetics and Data Science	Regeneron Genetics Center		
Eric	Jorgenson			Regeneron Genetics Center	Tarrytown, NY, USA	Analytical Genetics and Data Science	Regeneron Genetics Center		
Tyler	Joseph			Regeneron Genetics Center	Tarrytown, NY, USA	Analytical Genetics and Data Science	Regeneron Genetics Center		
Michael	Kessler			Regeneron Genetics Center	Tarrytown, NY, USA	Analytical Genetics and Data Science	Regeneron Genetics Center		
Jack	Kosmicki			Regeneron Genetics Center	Tarrytown, NY, USA	Analytical Genetics and Data Science	Regeneron Genetics Center		
Nan	Lin			Regeneron Genetics Center	Tarrytown, NY, USA	Analytical Genetics and Data Science	Regeneron Genetics Center		
Adam	Locke			Regeneron Genetics Center	Tarrytown, NY, USA	Analytical Genetics and Data Science	Regeneron Genetics Center		
Priyanka	Nakka			Regeneron Genetics Center	Tarrytown, NY, USA	Analytical Genetics and Data Science	Regeneron Genetics Center		
Jonathan	Marchini			Regeneron Genetics Center	Tarrytown, NY, USA	Analytical Genetics and Data Science	Regeneron Genetics Center		
Karl	Landheer			Regeneron Genetics Center	Tarrytown, NY, USA	Analytical Genetics and Data Science	Regeneron Genetics Center		
Olivier	Delaneau			Regeneron Genetics Center	Tarrytown, NY, USA	Analytical Genetics and Data Science	Regeneron Genetics Center		
Maya	Ghoussaini			Regeneron Genetics Center	Tarrytown, NY, USA	Analytical Genetics and Data Science	Regeneron Genetics Center		
Anthony	Marcketta			Regeneron Genetics Center	Tarrytown, NY, USA	Analytical Genetics and Data Science	Regeneron Genetics Center		
Joelle	Mbatchou			Regeneron Genetics Center	Tarrytown, NY, USA	Analytical Genetics and Data Science	Regeneron Genetics Center		
Arden	Moscati			Regeneron Genetics Center	Tarrytown, NY, USA	Analytical Genetics and Data Science	Regeneron Genetics Center		
Aditeya	Pandey			Regeneron Genetics Center	Tarrytown, NY, USA	Analytical Genetics and Data Science	Regeneron Genetics Center		
Anita	Pandit			Regeneron Genetics Center	Tarrytown, NY, USA	Analytical Genetics and Data Science	Regeneron Genetics Center		
Charles	Paulding			Regeneron Genetics Center	Tarrytown, NY, USA	Analytical Genetics and Data Science	Regeneron Genetics Center		
Jonathan	Ross			Regeneron Genetics Center	Tarrytown, NY, USA	Analytical Genetics and Data Science	Regeneron Genetics Center		
Carlo	Sidore			Regeneron Genetics Center	Tarrytown, NY, USA	Analytical Genetics and Data Science	Regeneron Genetics Center		
Eli	Stahl			Regeneron Genetics Center	Tarrytown, NY, USA	Analytical Genetics and Data Science	Regeneron Genetics Center		
Maria	Suciu			Regeneron Genetics Center	Tarrytown, NY, USA	Analytical Genetics and Data Science	Regeneron Genetics Center		
Timothy	Thornton			Regeneron Genetics Center	Tarrytown, NY, USA	Analytical Genetics and Data Science	Regeneron Genetics Center		

Supplemental Online Content: Nonauthor Collaborators

*Indicates required information. Only first name, last name, and suffix will appear in PubMed.

***First Name and Middle Initial(s)**	***Last Name**	***Suffix (eg, Jr, III)**	Academic Degrees	Institution	Location (city, state/province, country)	Role or Contribution, eg, chair, principal investigator	Group (if more than 1 Group listed in the byline) and/or Subgroup (eg, Steering Committee)		
Peter	VandeHaar			Regeneron Genetics Center	Tarrytown, NY, USA	Analytical Genetics and Data Science	Regeneron Genetics Center		
Sailaja	Vedantam			Regeneron Genetics Center	Tarrytown, NY, USA	Analytical Genetics and Data Science	Regeneron Genetics Center		
Scott	Vrieze			Regeneron Genetics Center	Tarrytown, NY, USA	Analytical Genetics and Data Science	Regeneron Genetics Center		
Jingning	Zhang			Regeneron Genetics Center	Tarrytown, NY, USA	Analytical Genetics and Data Science	Regeneron Genetics Center		
Rujin	Wang			Regeneron Genetics Center	Tarrytown, NY, USA	Analytical Genetics and Data Science	Regeneron Genetics Center		
Kuan-Han	Wu			Regeneron Genetics Center	Tarrytown, NY, USA	Analytical Genetics and Data Science	Regeneron Genetics Center		
Bin	Ye			Regeneron Genetics Center	Tarrytown, NY, USA	Analytical Genetics and Data Science	Regeneron Genetics Center		
Blair	Zhang			Regeneron Genetics Center	Tarrytown, NY, USA	Analytical Genetics and Data Science	Regeneron Genetics Center		
Andrey	Ziyatdinov			Regeneron Genetics Center	Tarrytown, NY, USA	Analytical Genetics and Data Science	Regeneron Genetics Center		
Yuxin	Zou			Regeneron Genetics Center	Tarrytown, NY, USA	Analytical Genetics and Data Science	Regeneron Genetics Center		
Olivier	Delaneau			Regeneron Genetics Center	Tarrytown, NY, USA	Analytical Genetics and Data Science	Regeneron Genetics Center		
Maya	Ghoussaini			Regeneron Genetics Center	Tarrytown, NY, USA	Analytical Genetics and Data Science	Regeneron Genetics Center		
Jingning	Zhang			Regeneron Genetics Center	Tarrytown, NY, USA	Analytical Genetics and Data Science	Regeneron Genetics Center		
Kyoko	Watanabe			Regeneron Genetics Center	Tarrytown, NY, USA	Analytical Genetics and Data Science	Regeneron Genetics Center		
Mira	Tang			Regeneron Genetics Center	Tarrytown, NY, USA	Analytical Genetics and Data Science	Regeneron Genetics Center		
Frank	Wendt			Regeneron Genetics Center	Tarrytown, NY, USA	Analytical Genetics and Data Science	Regeneron Genetics Center		
Adolfo	Ferrando			Regeneron Genetics Center	Tarrytown, NY, USA	Therapeutic Area Genetics	Regeneron Genetics Center		
Giovanni	Coppola			Regeneron Genetics Center	Tarrytown, NY, USA	Therapeutic Area Genetics	Regeneron Genetics Center		
Luca A	Lotta			Regeneron Genetics Center	Tarrytown, NY, USA	Therapeutic Area Genetics	Regeneron Genetics Center		
Alan	Shuldiner			Regeneron Genetics Center	Tarrytown, NY, USA	Therapeutic Area Genetics	Regeneron Genetics Center		
Katherine	Siminovitch			Regeneron Genetics Center	Tarrytown, NY, USA	Therapeutic Area Genetics	Regeneron Genetics Center		
Brian	Hobbs			Regeneron Genetics Center	Tarrytown, NY, USA	Therapeutic Area Genetics	Regeneron Genetics Center		
Jon	Silver			Regeneron Genetics Center	Tarrytown, NY, USA	Therapeutic Area Genetics	Regeneron Genetics Center		
William	Palmer			Regeneron Genetics Center	Tarrytown, NY, USA	Therapeutic Area Genetics	Regeneron Genetics Center		
Rita	Guerreiro			Regeneron Genetics Center	Tarrytown, NY, USA	Therapeutic Area Genetics	Regeneron Genetics Center		
Amit	Joshi			Regeneron Genetics Center	Tarrytown, NY, USA	Therapeutic Area Genetics	Regeneron Genetics Center		
Antoine	Baldassari			Regeneron Genetics Center	Tarrytown, NY, USA	Therapeutic Area Genetics	Regeneron Genetics Center		
Cristen	Willer			Regeneron Genetics Center	Tarrytown, NY, USA	Therapeutic Area Genetics	Regeneron Genetics Center		
Sarah	Graham			Regeneron Genetics Center	Tarrytown, NY, USA	Therapeutic Area Genetics	Regeneron Genetics Center		
Ernst	Mayerhofer			Regeneron Genetics Center	Tarrytown, NY, USA	Therapeutic Area Genetics	Regeneron Genetics Center		
Mary	Haas			Regeneron Genetics Center	Tarrytown, NY, USA	Therapeutic Area Genetics	Regeneron Genetics Center		
Niek	Verweij			Regeneron Genetics Center	Tarrytown, NY, USA	Therapeutic Area Genetics	Regeneron Genetics Center		
George	Hindy			Regeneron Genetics Center	Tarrytown, NY, USA	Therapeutic Area Genetics	Regeneron Genetics Center		
Jonas	Bovijn			Regeneron Genetics Center	Tarrytown, NY, USA	Therapeutic Area Genetics	Regeneron Genetics Center		
Tanima	De			Regeneron Genetics Center	Tarrytown, NY, USA	Therapeutic Area Genetics	Regeneron Genetics Center		
Parsa	Akbari			Regeneron Genetics Center	Tarrytown, NY, USA	Therapeutic Area Genetics	Regeneron Genetics Center		
Luanluan	Sun			Regeneron Genetics Center	Tarrytown, NY, USA	Therapeutic Area Genetics	Regeneron Genetics Center		
Olukayode	Sosina			Regeneron Genetics Center	Tarrytown, NY, USA	Therapeutic Area Genetics	Regeneron Genetics Center		
Arthur	Gilly			Regeneron Genetics Center	Tarrytown, NY, USA	Therapeutic Area Genetics	Regeneron Genetics Center		
Peter	Dornbos			Regeneron Genetics Center	Tarrytown, NY, USA	Therapeutic Area Genetics	Regeneron Genetics Center		
Juan	Rodriguez-Flores			Regeneron Genetics Center	Tarrytown, NY, USA	Therapeutic Area Genetics	Regeneron Genetics Center		
Moeen	Riaz			Regeneron Genetics Center	Tarrytown, NY, USA	Therapeutic Area Genetics	Regeneron Genetics Center		

*Indicates required information. Only first name, last name, and suffix will appear in PubMed.

***First Name and Middle Initial(s)**	***Last Name**	***Suffix (eg, Jr, III)**	Academic Degrees	Institution	Location (city, state/province, country)	Role or Contribution, eg, chair, principal investigator	Group (if more than 1 Group listed in the byline) and/or Subgroup (eg, Steering Committee)		
Manav	Kapoor			Regeneron Genetics Center	Tarrytown, NY, USA	Therapeutic Area Genetics	Regeneron Genetics Center		
Gannie	Tzoneva			Regeneron Genetics Center	Tarrytown, NY, USA	Therapeutic Area Genetics	Regeneron Genetics Center		
Momodou W	Jallow			Regeneron Genetics Center	Tarrytown, NY, USA	Therapeutic Area Genetics	Regeneron Genetics Center		
Anna	Alkelai			Regeneron Genetics Center	Tarrytown, NY, USA	Therapeutic Area Genetics	Regeneron Genetics Center		
Giovanni	Coppola			Regeneron Genetics Center	Tarrytown, NY, USA	Therapeutic Area Genetics	Regeneron Genetics Center		
Ariane	Ayer			Regeneron Genetics Center	Tarrytown, NY, USA	Therapeutic Area Genetics	Regeneron Genetics Center		
Veera	Rajagopal			Regeneron Genetics Center	Tarrytown, NY, USA	Therapeutic Area Genetics	Regeneron Genetics Center		
Sahar	Gelfman			Regeneron Genetics Center	Tarrytown, NY, USA	Therapeutic Area Genetics	Regeneron Genetics Center		
Vijay	Kumar			Regeneron Genetics Center	Tarrytown, NY, USA	Therapeutic Area Genetics	Regeneron Genetics Center		
Jacqueline	Otto			Regeneron Genetics Center	Tarrytown, NY, USA	Therapeutic Area Genetics	Regeneron Genetics Center		
Neelroop	Parikshak			Regeneron Genetics Center	Tarrytown, NY, USA	Therapeutic Area Genetics	Regeneron Genetics Center		
Aysegul	Guvenek			Regeneron Genetics Center	Tarrytown, NY, USA	Therapeutic Area Genetics	Regeneron Genetics Center		
Jose	Bras			Regeneron Genetics Center	Tarrytown, NY, USA	Therapeutic Area Genetics	Regeneron Genetics Center		
Silvia	Alvarez			Regeneron Genetics Center	Tarrytown, NY, USA	Therapeutic Area Genetics	Regeneron Genetics Center		
Jessie	Brown			Regeneron Genetics Center	Tarrytown, NY, USA	Therapeutic Area Genetics	Regeneron Genetics Center		
Jing	He			Regeneron Genetics Center	Tarrytown, NY, USA	Therapeutic Area Genetics	Regeneron Genetics Center		
Hossein	Khiabanian			Regeneron Genetics Center	Tarrytown, NY, USA	Therapeutic Area Genetics	Regeneron Genetics Center		
Joana	Revez			Regeneron Genetics Center	Tarrytown, NY, USA	Therapeutic Area Genetics	Regeneron Genetics Center		
Kimberly	Skead			Regeneron Genetics Center	Tarrytown, NY, USA	Therapeutic Area Genetics	Regeneron Genetics Center		
Valentina	Zavala			Regeneron Genetics Center	Tarrytown, NY, USA	Therapeutic Area Genetics	Regeneron Genetics Center		
Jae S	Sul			Regeneron Genetics Center	Tarrytown, NY, USA	Therapeutic Area Genetics	Regeneron Genetics Center		
Marcus B	Jones			Regeneron Genetics Center	Tarrytown, NY, USA	Research Program Management & Strategic Initiatives	Regeneron Genetics Center		
Esteban	Chen			Regeneron Genetics Center	Tarrytown, NY, USA	Research Program Management & Strategic Initiatives	Regeneron Genetics Center		
Michelle G	LeBlanc			Regeneron Genetics Center	Tarrytown, NY, USA	Research Program Management & Strategic Initiatives	Regeneron Genetics Center		
Jason	Mighty			Regeneron Genetics Center	Tarrytown, NY, USA	Research Program Management & Strategic Initiatives	Regeneron Genetics Center		
Nirupama	Nishtala			Regeneron Genetics Center	Tarrytown, NY, USA	Research Program Management & Strategic Initiatives	Regeneron Genetics Center		
Nadia	Rana			Regeneron Genetics Center	Tarrytown, NY, USA	Research Program Management & Strategic Initiatives	Regeneron Genetics Center		
Jennifer	Rico-Varela			Regeneron Genetics Center	Tarrytown, NY, USA	Research Program Management & Strategic Initiatives	Regeneron Genetics Center		
Jaimee	Hernandez			Regeneron Genetics Center	Tarrytown, NY, USA	Research Program Management & Strategic Initiatives	Regeneron Genetics Center		
Alison	Fenney			Regeneron Genetics Center	Tarrytown, NY, USA	Senior Partnerships & Business Operations	Regeneron Genetics Center		
Randi	Schwartz			Regeneron Genetics Center	Tarrytown, NY, USA	Senior Partnerships & Business Operations	Regeneron Genetics Center		

Supplemental Online Content: Nonauthor Collaborators

*Indicates required information. Only first name, last name, and suffix will appear in PubMed.

***First Name and Middle Initial(s)**	***Last Name**	***Suffix (eg, Jr, III)**	Academic Degrees	Institution	Location (city, state/province, country)	Role or Contribution, eg, chair, principal investigator	Group (if more than 1 Group listed in the byline) and/or Subgroup (eg, Steering Committee)		
Jody	Hankins			Regeneron Genetics Center	Tarrytown, NY, USA	Senior Partnerships & Business Operations	Regeneron Genetics Center		
Samuel	Hart			Regeneron Genetics Center	Tarrytown, NY, USA	Senior Partnerships & Business Operations	Regeneron Genetics Center		
Ann	Perez-Beals			Regeneron Genetics Center	Tarrytown, NY, USA	Business Operations & Administrative Coordinators	Regeneron Genetics Center		
Gina	Solari			Regeneron Genetics Center	Tarrytown, NY, USA	Business Operations & Administrative Coordinators	Regeneron Genetics Center		
Jaimee	Hernandez			Regeneron Genetics Center	Tarrytown, NY, USA	Business Operations & Administrative Coordinators	Regeneron Genetics Center		
Johannie	Rivera-Picart			Regeneron Genetics Center	Tarrytown, NY, USA	Business Operations & Administrative Coordinators	Regeneron Genetics Center		
Michelle	Pagan			Regeneron Genetics Center	Tarrytown, NY, USA	Business Operations & Administrative Coordinators	Regeneron Genetics Center		
Sunilbe	Siceron			Regeneron Genetics Center	Tarrytown, NY, USA	Business Operations & Administrative Coordinators	Regeneron Genetics Center		
Adam	Buchanan			Geisinger Health System	Danville, PA, USA		The Geisinger MyCode Community Health Initiative		
David J.	Carey			Geisinger Health System	Danville, PA, USA		The Geisinger MyCode Community Health Initiative		
Christa L.	Martin			Geisinger Health System	Danville, PA, USA		The Geisinger MyCode Community Health Initiative		
Michelle	Meyer			Geisinger Health System	Danville, PA, USA		The Geisinger MyCode Community Health Initiative		
Kyle	Retterer			Geisinger Health System	Danville, PA, USA		The Geisinger MyCode Community Health Initiative		
David	Rolston			Geisinger Health System	Danville, PA, USA		The Geisinger MyCode Community Health Initiative		
