# Supplementary material for: Rare and Common Genetic Variation Underlying Atrial Fibrillation Risk
Source: JAMA Cardiol. 2024 Jun 26;9(8):732–40. doi: 10.1001/jamacardio.2024.1528 (PMC11209175; doi:10.1001/jamacardio.2024.1528)
Supplement: Supplement 4. — Data sharing statement [file jamacardiol-e241528-s004.pdf]

Data Sharing Statement

Vad. Rare and Common Genetic Variation Underlying Atrial Fibrillation Risk. *JAMA Cardiol*. Published June 26, 2024. doi:10.1001/jamacardio.2024.1528

Data
Data available: No

Additional Information
Explanation for why data not available: We are not able to make individual level data available. Data from the UK Biobank supporting this manuscript (including individual level data) is available for bona-fide researchers through application to the UK Biobank.
